# Towards an understanding of the propensity for crystalline hydrate formation by molecular compounds

**DOI:** 10.1107/S2052252516015633

**Published:** 2016-10-18

**Authors:** Alankriti Bajpai, Hayley S. Scott, Tony Pham, Kai-Jie Chen, Brian Space, Matteo Lusi, Miranda L. Perry, Michael J. Zaworotko

**Affiliations:** a Department of Chemical Sciences, Bernal Institute, University of Limerick, Co. Limerick, Ireland; b Department of Chemistry, CHE 205, University of South Florida, 4202 East Fowler Avenue, Tampa, Florida 33620, USA

**Keywords:** molecular hydrates, CSD survey, hydrate screening experiments, electrostatic potential, hydrogen bonding, *N*-heterocyclic aromatic compounds

## Abstract

Hydrates are technologically important and ubiquitous yet they remain a poorly understood and understudied class of molecular crystals. In this work, we attempt to rationalize propensity towards hydrate formation through crystallization studies of molecules that lack strong hydrogen-bond donor groups. A Cambridge Structural Database (CSD) survey indicates that the statistical occurrence of hydrates in 124 molecules that contain five- and six-membered *N*-heterocyclic aromatic moieties is 18.5%. However, hydrate screening experiments on a library of 11 *N*-heterocyclic aromatic compounds with at least two acceptor moieties and no competing hydrogen-bond donors or acceptors reveals that over 70% of this group form hydrates, suggesting that extrapolation from CSD statistics might, at least in some cases, be deceiving. Slurrying in water and exposure to humidity were found to be the most effective discovery methods. Electrostatic potential maps and/or analysis of the crystal packing in anhydrate structures was used to rationalize why certain molecules did not readily form hydrates.

## Abstract 2

The propensity for crystalline hydrate formation by molecular compounds that are devoid of strong hydrogen-bond donors has been analyzed and rationalized through a Cambridge Structural Database (CSD) survey, systematic hydrate screening experiments and computational studies.

## 1. Introduction   

Hydrates represent a type of multicomponent crystals that are ubiquitous thanks to the presence of moisture in most crystallization reactions. That there is general interest in hydrates is reflected in the increasing number of publications on crystalline hydrates in areas such as pharmaceutical and materials sciences. Indeed, water is the most common solvent included in molecular crystals even if present adventitiously (Desiraju, 1991 ▸; Görbitz & Hersleth, 2000 ▸). The existence of hydrates has been rationalized based on the following features of a water molecule: (i) small size; (ii) tendency to form multidirectional hydrogen bonds with itself as well as other compounds; (iii) ability to serve as a donor and/or acceptor for up to two hydrogen bonds (Desiraju, 1991 ▸). The study of hydrates is of particular significance in the context of the pharmaceutical industry (Khankari & Grant, 1995 ▸; Vippagunta *et al.*, 2001 ▸; Trask *et al.*, 2006 ▸; Eddleston *et al.*, 2014 ▸; Madusanka *et al.*, 2014 ▸) since hydrate formation can alter the physicochemical properties of a drug substance, sometimes positively (Morris, 1999 ▸). Indeed, a hydrate is the selected solid form for several commercial drug substances (Lee *et al.*, 2011 ▸), *e.g.* cefadroxil (monohydrate) (Bouzard *et al.*, 1985 ▸), paroxetine hydrochloride (hemihydrate) (Barnes *et al.*, 1988 ▸), cephalexin (monohydrate) (Horatius, 1975 ▸), ampicillin (trihydrate) (Bahal, 1975 ▸), cromolyn sodium (disodium cromoglycate, non-stoichiometric hydrates) (Chen *et al.*, 1999 ▸) and nitrofurantoin (monohydrate) (Cazer *et al.*, 1994 ▸). Further, up to 33% of entries in the European Pharmacopeia (1991) are reported to exist as hydrates (Henck *et al.*, 1997 ▸). Water, being a non-toxic solvent, does not typically raise any serious regulatory concerns when it is present in a drug substance. However, in materials science, its presence, be it in trace amounts or in larger quantities, can affect the outcome of a reaction and/or negatively impact stability or performance. For example, many metal–organic materials (MOMs) degrade in the presence of water vapor (Ming *et al.*, 2015 ▸). Further, water vapor can diminish the gas sorption performance of physisorbents (Kumar *et al.*, 2015 ▸). However, this does not mean that the existence of hydrates is predictable or well understood. Indeed, we have suggested that the promiscuity of water makes it a nemesis of crystal engineering (Clarke *et al.*, 2010 ▸).

From a crystal engineering (Pepinsky, 1955 ▸; Schmidt, 1971 ▸; Desiraju, 1989 ▸; Moulton & Zaworotko, 2001 ▸) perspective, hydrates raise the following questions, amongst others: (i) Can one pre-determine whether or not a given organic compound is predisposed to form hydrate(s)? (ii) In general, how common is hydrate formation for molecular organic compounds? (iii) What are the most effective experimental methods for the discovery of hydrates? Several research groups have examined the statistical frequency of occurrence of crystalline hydrates (Infantes *et al.*, 2007 ▸), conditions for their formation (Khankari & Grant, 1995 ▸; Morris, 1999 ▸; Infantes *et al.*, 2007 ▸) and preferred chemical environments for water molecules (Gillon *et al.*, 2003 ▸; Infantes *et al.*, 2003 ▸; Hickey *et al.*, 2007 ▸). Statistical analyses typically rely on the Cambridge Structural Database (CSD) (Allen & Kennard, 1993 ▸; Allen, 2002 ▸), which contains almost one million structures and is a broad enough dataset for some if not most statistical studies. However, as we discuss herein the CSD is not a panacea for all queries related to crystal engineering. Additionally, software-based limitations are a general concern (Infantes & Motherwell, 2002 ▸; Mascal *et al.*, 2006 ▸; van de Streek & Motherwell, 2007 ▸). In particular, the types of crystal structures in the CSD can only serve as a backwards leaning representation of experimental outcomes, *i.e.* they are reflective of the types of molecules that were of interest in the past and are not necessarily representative of the full diaspora of molecular compounds. Further, with respect to hydrates in particular, the crystal structures reported in the CSD are not necessarily a result of systematic experiments aimed at hydrates and the CSD lacks experimental details about crystallization. For example, systematic screening experiments aimed at hydrates such as those undertaken routinely in pharmaceutical science (Grant & Higuchi, 1990 ▸; Griesser, 2006 ▸; Guillory, 1999 ▸; Morris, 1999 ▸; Zhu & Grant, 1996 ▸; Zhu, 1996 ▸) are rarely reported in the scientific literature (Newman & Wenslow, 2016 ▸).

Solvates, including hydrates, have been classified as two main types: stoichiometric and non-stoichiometric (Griesser, 2006 ▸). Based on their structural attributes, hydrates have been further classified into three categories: (i) channel hydrates; (ii) isolated site hydrates; (iii) metal ion associated hydrates (Morris & Rodriguez-Hornedo, 1993 ▸). As far as stoichiometric hydrates are concerned, attempts have been made to provide a rational basis for incorporation of water of hydration in crystals. Hypotheses such as propensity being linked to imbalance in the ratio of hydrogen-bond donors and acceptors (Desiraju, 1991 ▸) or the sum of and/or difference in the total number of hydrogen-bond donors and acceptors (Infantes *et al.*, 2007 ▸) have been advanced. These are largely in accordance with Etter’s hydrogen-bonding rule, which states that ‘all good proton donors and acceptors are used in hydrogen bonding’ (Etter, 1990 ▸). In this context, the identification of eight different environments for water by Gillon *et al.* is noteworthy (Gillon *et al.*, 2003 ▸). Most frequently, water serves as a donor of two hydrogen bonds and an acceptor of one hydrogen bond, and it has been suggested that the water environment correlates with the ratio of hydrogen-bond donors/acceptors in the molecular compound studied (Infantes *et al.*, 2007 ▸). Incorporation of water molecules in the crystal lattice is presumed to provide alternative modes of crystal packing through water-mediated supramolecular heterosynthons (Clarke *et al.*, 2010 ▸; Walsh *et al.*, 2003 ▸). Computational and statistical models have also been used to rationalize hydrate formation (Hulme & Price, 2007 ▸; Price, 2008 ▸; Braun *et al.*, 2011 ▸; Takieddin *et al.*, 2016 ▸). Electrostatic potential has been shown to be an effective indicator to predict the hydrate propensity in certain types of molecules (Murray *et al.*, 1991 ▸; Murray & Politzer, 1991 ▸; Galabov *et al.*, 2003 ▸). These studies have thus far been limited to specific molecules. However, despite much progress, the formation of hydrates still remains largely unpredictable and represents a challenge in crystal engineering (Clarke *et al.*, 2010 ▸).

**Figure 1:**
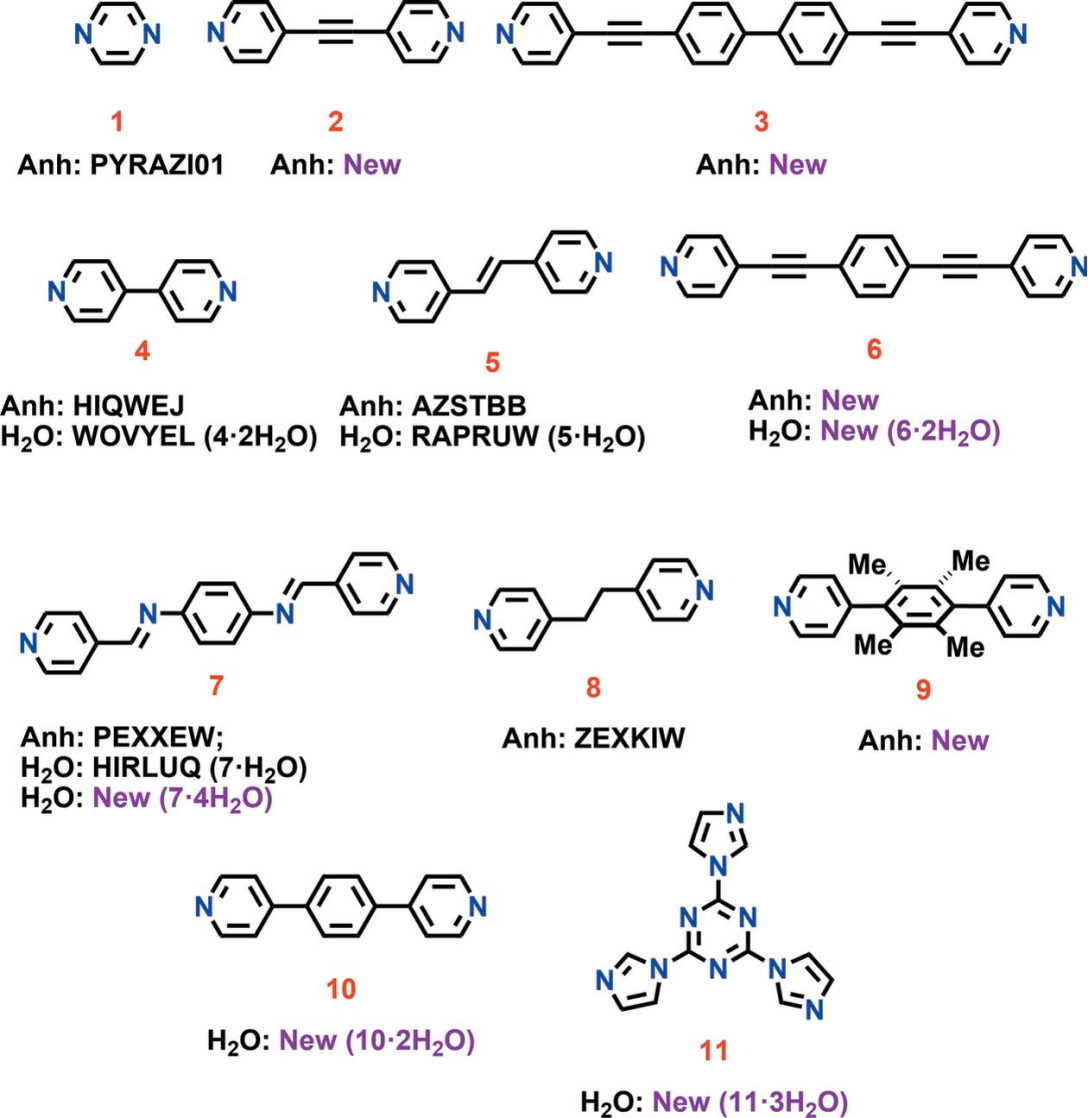
The library of *N*-heterocyclic compounds investigated herein for hydrate formation. Refcodes for those anhydrates (Anh) and hydrates (H_2_O) reported in the CSD are given. Previously unreported structures are denoted as ‘New’. The stoichiometry of water in hydrated structures is given in parentheses.

In previous reports, we demonstrated that a tetrafunctional molecular cluster, [{*M*(CO)_3_(μ_3_-OH)}_4_] (*M* = Mn or Re), can serve as a strong hydrogen-bond donor to form solvates, hydrates or cocrystals (Clerk & Zaworotko, 1991 ▸; Copp *et al.*, 1992 ▸, 1993 ▸, 1995 ▸), even with molecules that serve as only weak hydrogen-bond acceptors such as arenes (Copp *et al.*, 1995 ▸). Notably, [{*M*(CO)_3_(μ_3_-OH)}_4_] can only be crystallized from solution as a single-component crystal if a dried distilled solvent such as CHCl_3_ is used (Holman & Zaworotko, 1995 ▸). This is unsurprizing in the context of a subsequent CSD analysis, which suggested that unsatisfied hydrogen-bond donors might be the main driving force for hydrate formation (Infantes *et al.*, 2003 ▸). The corollary of this is that a high ratio of hydrogen-bond donors/acceptors would be expected to result in high propensity to form hydrates, as has been suggested by van de Streek and Motherwell (van de Streek & Motherwell, 2007 ▸). In this contribution, we examine the propensity for hydrate formation at one extreme through a CSD and experimental study. Specifically, we focus upon five- and six-membered *N*-heterocyclic aromatic compounds that contain two or more hydrogen-bond acceptors but are devoid of strong hydrogen-bond donors. Our experimental data were collected for a library of 11 molecular compounds (Fig. 1 ▸).

## 2. Experimental   

### 2.1. General aspects   

All reagents and solvents were purchased from Sigma–Aldrich, Alfa Aesar or AK scientific, and used as received. ^1^H and ^13^C Nuclear Magnetic Resonance (NMR) spectra were recorded on a Jeol EX270. Powder X-ray diffraction (PXRD) data were collected using a Philips X’Pert PRO MPD equipped with a Cu *K*α source. Data were collected from 5 to 40° 2θ, using a step size of 0.02° at a scan rate of 0.1° min^−1^. Thermogravimetric Analyses (TGA) were measured on a TA Instruments Q50 TG from ambient temperature to 500°C under a 60 ml min^−1^ flow of N_2_, at a scan rate of 20°C min^−1^. Karl Fisher titrations (volumetric) were performed on a Mettler DL31; Fisher Aqua­line, Solvent K/2100/15 and titrant K/2000/15 were used as two- and single-component reagents, respectively. Karl Fischer titrations were performed at 15–20% R.H. and 27°C.

### 2.2. X-ray crystallography   

X-ray diffraction data for **2**, **6**·2H_2_O and **9** were collected at 100 (2) K, while data for **7**·4H_2_O were collected at 273 (2) K, under N_2_ flow, on a Bruker Quest D8 Mo Sealed Tube equipped with CMOS camera and Oxford cryosystem with Mo *K*α radiation (λ = 0.71073 Å). Data for **3**, **6**, **10**·2H_2_O and **11**·3H_2_O were collected at 100 (2) K, under N_2_ flow, on a Bruker Quest D8 Cu Microfocus with Cu *K*α radiation (λ = 1.5418 Å). Indexing and data reduction were conducted using the Bruker *APEX*2 suite (Bruker, 2010 ▸) (Difference Vectors method) and corrected for absorption using the multi-scan method implemented in Bruker *SADABS* software (Sheldrick, 2008*b* ▸). All structures were solved by direct methods (*SHELXS*97), and refined (*SHELXL*97) by full least-squares on all *F* ^2^ data (Sheldrick, 2008*a* ▸; Spek, 1990 ▸). All non-H atoms were refined anisotropically. H atoms were placed in calculated positions, with the exception of those on water molecules, which were refined after location from inspection of the electron density map. In **11**·3H_2_O the H atoms of some of the water molecules could not be located and the O atoms were refined as isolated atoms. Crystallographic data and refinement parameters for all structures are given in Table 1 ▸.

### 2.3. Syntheses of compounds   

Compounds **1**, **4**, **5** and **8** were purchased from Sigma Aldrich and used as received. Compounds **2**, **3** and **6** were prepared by Pd^0^-catalyzed Sonogashira coupling of 4-ethynylpyridyine hydrochloride with the corresponding mono- or diiodo derivatives (4-iodopyridine, 1,4-diiodobenzene and 4,4′-diiodobiphenyl, respectively). Compound **7** was synthesized by condensation of 4-pyridylcarboxaldehyde and 1,4-diaminobenzene by refluxing in dry EtOH based on a procedure reported in the literature (Sek *et al.*, 2013 ▸). Compounds **9** and **10** were synthesized by twofold Pd^0^-catalyzed Suzuki cross-coupling of 4-pyridynylboronic acid with 1,4-dibromobenzene and 1,4-dibromodurene, respectively. Compound **11** was obtained by following a previously reported nucleophilic substitution on cyanuric chloride with imidazole under solvent free conditions (Azarifar *et al.*, 2004 ▸). The molecular structure of each compound was confirmed by ^1^H and ^13^C NMR spectroscopies and SCXRD. Detailed accounts of the syntheses of compounds **2**, **3**, **6**, **9**, and **10** are given in the supporting information.

### 2.4. Single crystals   

**1,2-Bis(4-pyridyl)acetylene (2).** Prism-shaped crystals of **2** were obtained *via* slow evaporation of a solution of **2** (20 mg, 0.11 mmol) in 4 ml of toluene over 3 d (yield 19 mg).

**4,4′-Bis(4-ethynylpyridyl)biphenyl (3).** Needle-shaped crystals of **3** were obtained by slow evaporation of a solution of **3** (25 mg, 0.07 mmol) in 5 ml of MeOH over 5 d (yield 15 mg).

**1,4-Bis(4-ethynylpyridyl)benzene (6).** Plate-shaped crystals of the anhydrous form of **6** were obtained by slow evaporation of a solution of **6** (30 mg, 10.7 mmol) in 4 ml of toluene over 10 d (yield 20 mg).

**1,4-Bis(4-ethynylpyridyl)benzene (6·2H_2_O).** Cubic crystals of the dihydrate of **6** were obtained by slow evaporation of a solution of **6** (30 mg, 10.7 mmol) in 4 ml of MeOH/H_2_O mixture (1:1 *v*/*v*) over 6 d (yield 12 mg).

**Bis(pyridin-4-yl­methylene)benzene-1,4-diamine tetrahydrate (7·4H_2_O).** Plate-like crystals of the tetrahydrate of **7** were obtained by slow evaporation of a saturated solution of **7** (30 mg, 0.10 mmol) in 5 ml of ethanol over a period of 5 d (yield 8 mg).

**1,4-Bis(4-pyridyl)­durene (9).** Cubic crystals of **9** were obtained by slow evaporation of a solution of **9** (25 mg, 0.09 mmol) in 5 ml of EtOH over 2 weeks (yield 19 mg).

**1,4-Bis(4-pyridyl)benzene dihydrate (10·2H_2_O).** Needle-shaped crystals of **10·2H_2_O** were obtained by slow evaporation of a solution of **10** (25 mg, 0.11 mmol) in 5 ml of ethyl acetate over 5 d (yield 24 mg).

**2,4,6-Tris(imidazol-1-yl)-1,3,5-*s*-triazine (11·3H_2_O).** Column-shaped crystals of the trihydrate of **11** were obtained by slow evaporation of **11** (30 mg, 0.11 mmol) in 3 ml of ethyl acetate over 5 d (yield 12 mg).

### 2.5. Slurry experiments   

50 mg of compound was slurried in a solvent system acceptable for use in the pharmaceutical industry in a sealed glass vial at room temperature. The volume of solvent used was one-third of the volume required to dissolve the sample completely. Aliquots of sample were removed after 1, 2, 4, 5 and 7 d in order to record the PXRD patterns.

### 2.6. Stability   

To determine stability under ambient conditions, samples were exposed to the laboratory atmosphere. PXRD data were recorded after 1, 3, 7, 10 and 30 d. Stability to humidity was evaluated by placing 50 mg of each sample in a humidity chamber under 75% relative humidity (R.H.) at 40°C. Aliquots were removed from the chamber after 6, 7, 10, 12 and 14 d and PXRD data were collected on each aliquot.

### 2.7. Solvent drop grinding (SDG)   

20 mg of anhydrous sample was placed in an agate mortar and 10 µL of water was added. Mild hand grinding with a pestle was conducted until the initial paste became a fine powder (*ca.* 10 min). The sample was then characterized by PXRD and TGA.

### 2.8. Electrostatic potential map calculations   

The atomic positions of the molecules of compounds **1**–**11** were fully optimized using second-order Møller–Plesset perturbation theory (MP2) (Møller & Plesset, 1934 ▸) with the 6-31G* basis set applied to all atoms. The optimization calculations were performed with the NWChem *ab initio* simulation software (Valiev *et al.*, 2010 ▸). For each compound, a three-dimensional surface around the molecule was calculated, where the electron density was equal to 0.002 a.u. The resulting isodensity surface served as the basis for mapping the electrostatic potential. The electrostatic potential of the respective molecules was then calculated using density functional theory (DFT) with the 6-31G* basis set for all atoms and with the M06 hybrid functional (Zhao & Truhlar, 2008 ▸). A graphical representation (map) of the electrostatic potential surface for each molecule was generated using *Spartan* ’14 software (Wavefunction, 2014 ▸).

## 3. Results and discussion   

### 3.1. CSD analysis   

The CSD (ConQuest 1.18, CSD v5.37 + 1 November 2015 update (Bruno *et al.*, 2002 ▸), only organics, three-dimensional coordinates determined and *R* ≤ 0.075; Group I and II elements were excluded) contains 257 442 entries that would be classified as molecular organic crystal structures. Of these, 16 710 (6.5%) were found to contain water molecules. We focused our analysis upon molecules containing only hydrogen-bond acceptors, specifically five- and six-membered *N*-heterocyclic aromatic rings: pyridyl, pyrimidyl, pyrrolyl and *R*-imidazoyl moieties, Fig. S1. Compounds with *ortho*-substituents were excluded to eliminate any bias caused by steric effects. A total of 4962 hits were retrieved as *hitlist* 1 (Fig. S1). Of these, 564 entries (11.4%, *hitlist* 2) contain one or more water molecules and 288 (*hitlist* 3) contain a neutral organic molecule and at least one water molecule in the asymmetric unit. The remaining 276 entries are multicomponent systems containing water. In *hitlist* 3, water molecules were observed to most commonly hydrogen bond to aromatic nitrogen atoms (197 entries). Other moieties found to interact with water molecules include amido (98 entries), primary and secondary amino (29 entries), hydroxyl (10 entries) and/or carboxyl groups (7 entries).

*Hitlist* 1 (4962 entries) was further restricted by excluding molecular compounds containing competing hydrogen-bond donor and acceptor groups (Fig. S1). Entries with hydrogen-bond donors such as primary and secondary amino, imino, hydroxyl, carboxyl, hydroxysulfonyl and thio were thereby excluded. Likewise, compounds with hydrogen-bond acceptors such as amido, imino, hydroxyl, carboxyl, alkoxy, alkoxy­carbonyl/aryloxycarbonyl, carbonyl, nitro, cyano and halo were excluded, resulting in *hitlist* 4 (482 entries). *Hitlist* 4 was examined manually and those entries containing alkyl chains with more than three C atoms were eliminated, as were those with fused aromatic rings larger or equivalent in size to anthracene/phenanthrene. These exclusions addressed the potential influence of large aliphatic/aromatic moieties on crystal packing. The number of entries was thereby reduced to 139 (*hitlist* 5). To determine how many individual organic molecules remained, we removed duplicates, polymorphs and hydrates. At this point, 124 unique compounds remained (*hitlist* 6), of which 23 (or 18.5%) are known to form hydrates (*hitlist* 7).

The above statistics indicate that the propensity to form hydrates for this class of hydrogen-bond acceptors is impacted by competing hydrogen-bonding functional groups: *ca.* 11.4% of the hits are hydrates in a competitive environment whereas *ca.* 18.5% of the entries are hydrates in a non-competitive environment. The propensity for hydrate formation discerned from our statistical analysis using the refined data correlates well with that previously reported for molecular compounds with *sp* ^2^-hybridized nitrogen atoms (*ca*. 16.8%) (Infantes *et al.*, 2003 ▸). However, is 16.8% an indication of the general propensity for this class of compounds? In order to address this matter we conducted a series of hydrate screening experiments.

### 3.2. Hydrate screening experiments   

We selected a library of five- and six-membered *N*-heterocyclic aromatic compounds, developed as part of our ongoing research into crystal engineering of MOMs and hybrid ultramicroporous materials (Subramanian & Zaworotko, 1995 ▸; Burd *et al.*, 2012 ▸; Nugent *et al.*, 2013*a* ▸,*b* ▸; Scott *et al.*, 2015 ▸; Chen *et al.*, 2015 ▸; Cui *et al.*, 2016 ▸), as representative of molecules which contain only strong hydrogen-bond acceptors, Fig. 1 ▸. Molecules **1**–**3**, **6** and **9** are linear diaza compounds with rigidity imparted as a consequence of electronic and steric factors. Compounds **4** and **10** can exhibit torsional flexibility about the σ-bond between the two pyridyl rings. Dipyridylethylene **5**, diimine **7** and dipyridylethane **8** likewise exhibit conformational flexibility. Trisimidazolyltriazine **11** contains six potential hydrogen-bond acceptors and considerable torsional flexibility. These structurally and electronically related compounds were subjected to the following hydrate screening experiments: (i) crystallization from mixed solvent systems; (ii) slurrying in water at ambient temperature; (iii) exposure of anhydrous powders to humid conditions; (iv) solvent drop grinding (SDG). The hydrate screening experiments are collated in Table 2 ▸ and presented as a flowchart in Fig. S2.

Crystallization from mixed solvent systems afforded eight new single-crystal structures of which four are hydrates (compounds **6**, **7**, **10** and **11**, as shown in Figs. 1 ▸ and 2 ▸). Method (i) therefore afforded hydrates at a greater frequency than suggested by our CSD survey. Further details and analyses of the crystal structures are provided in the following section and in the supporting information.

Slurry experiments using water or water/organic solvent mixtures have previously been used to screen for the existence of hydrates (Cui & Yao, 2008 ▸; Ticehurst *et al.*, 2002 ▸). Beginning with pure water, slurries of **1**–**11** were performed under ambient conditions. PXRD and thermogravimetric analyses (TGA) were used to determine that hydrates were isolated for **4**–**11**. To examine the relative stability of the isolated hydrates, the anhydrous and hydrated forms in 1:1 *w*/*w* ratios were slurried in mixed solvent systems with varying ratios of EtOH and H_2_O. The presence of water in a solvent mixture in a fivefold excess of EtOH invariably afforded hydrates, see the supporting information.

Standard accelerated stability testing conditions as required in the pharmaceutical industry (40°C and 75% relative humidity (R.H.)) (Huynh-Ba, 2008 ▸) were employed for **2**–**11**. Pyrazine (**1**) was not subjected to these conditions as it sublimes under ambient conditions. 4,4′-Dipyridyl (**4**) was studied first since both the anhydrous and hydrated forms of **4** were previously reported (Boag *et al.*, 1999 ▸; Näther *et al.*, 2001 ▸). We observed a gradual transition of the anhydrous form into the hydrated form over a period of 7 d as determined by PXRD, Fig. S18. Anhydrous forms of the remaining compounds were subsequently exposed to 75% R.H. at 40°C for a minimum of 7 d, or until complete conversion had occurred. Hydrates of **4**–**11** were isolated under these conditions and their PXRD patterns were found to match those from the slurry experiments. The presence of water in the samples obtained from humidity exposure was verified by PXRD and weight loss corresponding to the appropriate amount of water in TGA (supporting information).

Anhydrous variants of **1**–**11** were also subjected to aqueous solvent drop grinding (Karki *et al.*, 2007 ▸; Shah & Amidon, 2014 ▸) (method iv). **1**–**3** did not form a hydrate after mild hand grinding for 30 min. **4** and **8** were isolated as dihydrate and anhydrate, respectively (Figs. S16 and S32). Other compounds were isolated as mixtures of both forms (supporting information). Solvent-drop grinding experiments with pyrazine **1** could not be performed as it sublimes at room temperature.

To gauge the stability of the hydrates of **4**–**8**, **10** and **11**, the samples were exposed to ambient laboratory conditions. PXRD studies revealed that the hydrates of **4**, **6** and **11** were found to be stable for 30 d, whereas those of **7**, **8** and **10** converted to anhydrous forms within 1 d. The hydrate of **5** retained its stability for 10 d, but it started to convert to its anhydrous form within 30 d.

The thermal stability of hydrates of **4**–**7**, **10** and **11** was evaluated by TGA (supporting information). The temperature at which water is lost was analyzed and compared with structural attributes such as type of hydrate (channel or isolated site hydrate), number of hydrogen-bond donors and acceptors and hydrogen-bond distances to determine any correlation. For **5**·H_2_O, an isolated site hydrate, the loss of water occurs above 100°C, suggesting that water molecules are tightly bound. For **7**·4H_2_O, water loss occurred below 100°C. For those channel hydrates in which water molecules are organized into one-dimensional chains (**4**·2H_2_O, **6**·2H_2_O and **10**·2H_2_O) and tapes (**11**·3H_2_O), loss of water was observed to occur below 100°C. These observations are consistent with our previous findings concerning structure/stability of cocrystal hydrates (Clarke *et al.*, 2010 ▸).

Overall, the hydrate screening experiments revealed that 8 out of 11 (72.7%) of the molecules studied form hydrates, a much greater propensity than suggested by our CSD survey. Slurrying anhydrous forms in water under ambient conditions was the most effective method to isolate hydrates (**4**–**11**).

### 3.3. Analysis of water clusters   

**Figure 2:**
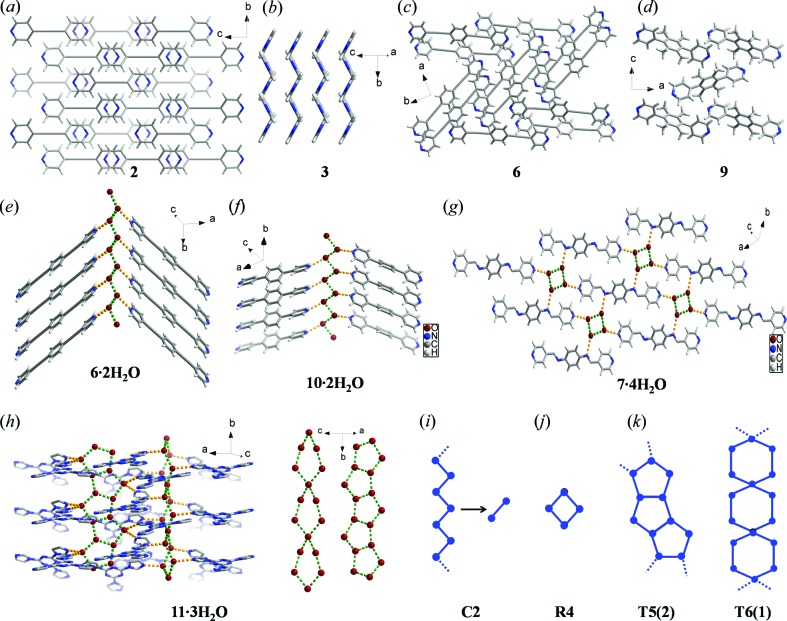
New crystal structures of (*a*)–(*d*) anhydrous and (*e*)–(*h*) hydrated forms of *N*-heterocyclic aromatics **1**–(11). (*i*)–(*k*) Water molecules organize into (*i*) one-dimensional *infinite chains* (C2) in **6**·2H_2_O and **11**·2H_2_O, (*j*) *discrete rings* (*R*4) in **7**·4H_2_O, and (*k*) pentagonal (*T*5(2)) and hexagonal *infinite tapes* (*T*6(1)) in **11**·3H_2_O.

**Figure 3:**
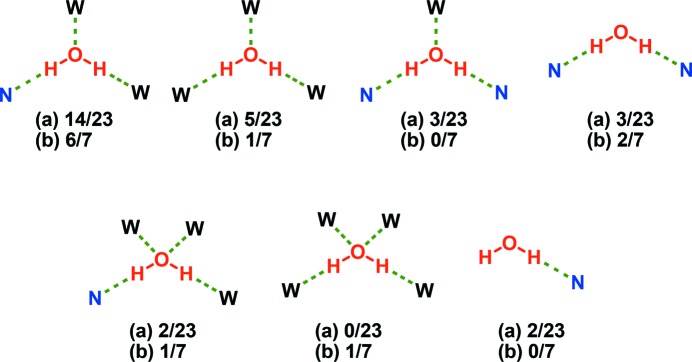
Patterns in which water molecules are hydrogen bonded to water (W) and/or *N*-heterocyclic rings (N) in (*a*) the 23 structures retrieved from the CSD search and (*b*) 7 structures included for screening experiments.

The hydrogen-bond environments of water molecules were analyzed in the 23 hydrates obtained from our CSD analysis (*hitlist* 7) and the seven hydrates isolated herein (Fig. 3 ▸). In both subsets, water molecules tend to exhibit two hydrogen-bond donors and one hydrogen-bond acceptor, which is consistent with previous findings (Infantes *et al.*, 2007 ▸). Crystal structure analysis reveals no disorder and no particularly significant thermal motion for the water O atoms at the experimental temperature as judged by their thermal displacement parameters. The stoichiometry of water in the crystal lattice seems to affect what water clusters or hydrogen-bond patterns are present in these subsets. Infantes *et al.* have classified water clusters as discrete rings and chains, infinite chains and tapes and layer structures and assigned the symbols R, D, C, T and L, respectively. These symbols were further refined by suffix ‘n’, specifying the number of water molecules forming the repeat unit. For example, ‘C2’ means the water molecules form one-dimensional infinite chains, and *n* = 2 signifies two water molecules form the unit cell repeat unit of the chain or one crystallographically independent water molecule is repeated by a *C*2-screw axis, Fig. 2 ▸(*i*). The authors also calculated the frequency of occurrence of each of these water clusters in crystal structures reported in the CSD (Infantes & Motherwell, 2002 ▸; Infantes *et al.*, 2003 ▸). We have adopted this nomenclature herein. In general, dihydrates occur for dipyridyls whereas trihydrates tend to be formed by tri­pyridyls. Out of the 23 hydrates in *hitlist* 7, six are dihydrates and five of these contain C2 chains. Six of the 23 hydrates are trihydrates but their water clusters vary. Only one entry is a tetrahydrate with a *T*4(2) water cluster. If we include **1**–**11**, the number of dihydrates increases to eight (**6**·2H_2_O and **10**·2H_2_O) and 7/8 contain C2 chains, Fig. 2 ▸(*i*). The stoichiometry as determined from TGA and Karl Fisher titration experiments conducted upon the hydrates of **8** and **9** is inconsistent with dihydrates (supporting information). This might be attributed to conformational flexibility and torsional rigidity in **8** and **9**, respectively. As a result, assembly into C2 chains, which requires molecules to be in close proximity to each other, is hindered. The number of tri- and tetrahydrates increases by one each because of **11**·3H_2_O and **7**·4H_2_O, respectively. **11**·3H_2_O exhibits infinite pentagonal (*T*5(2)) and hexagonal (*T*6(1)) tapes (Fig. 2 ▸ *k*), whereas **7**·4H_2_O forms *R*4 clusters (Fig. 2 ▸ *j*). We note that association of water molecules into one-dimensional chains and/or tapes facilitates *N*-heterocyclics to further associate through π–π (face-to-face) interactions (Table S1). As a result, O—H⋯N and π–π (face-to-face) interactions are dominant in their crystal packing.

### 3.4. Computational studies   

**Figure 4:**
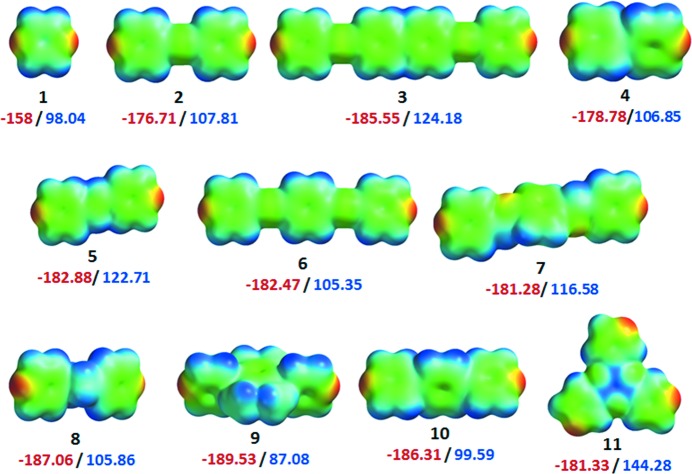
The electrostatic potential maps (kJ mol^−1^) for *N*-heterocyclic aromatics **1**–**11**.

The results of the hydrate screening experiments reported herein indicate that molecules with similar functionality can behave quite differently with respect to hydrate formation. This is unsurprising given previous studies upon hydrates and begs the following question: what makes **1**–**3** behave differently than **4**–**11**? The molecular electrostatic potential has been shown to serve as an effective tool for correlating with and even predicting molecular interactions and crystal behaviour (Scrocco & Tomasi, 1978 ▸; Politzer & Daiker, 1981 ▸; Politzer & Murray, 1991 ▸). Politzer and co-workers have shown that the ability of a solute molecule to accept or donate a proton in solution (solvatochromic hydrogen-bond donor and acceptor parameters) can be correlated with its calculated electrostatic potential, which pertains to the molecule in its gas phase (Murray *et al.*, 1991 ▸; Murray & Politzer, 1991 ▸). Later, Galabov *et al.* (2003 ▸) have also shown that there exist excellent linear relationships between molecular electrostatic potentials of the nuclei participating in hydrogen bonding and the binding energies. These studies exemplify that calculated electrostatic potential can be used as an indicator to predict the hydrate propensity in certain types of molecules. Electrostatic potentials were calculated for **1**–**11** and mapped on the molecular electron density surfaces (Fig. 4 ▸) in an attempt to correlate electrostatic potential at the nitrogen atom with propensity for hydrate formation. **1** is an outlier since its nitrogen atoms are calculated to have a much lower electrostatic potential energy (−158 kJ mol^−1^) than **2**–**11** (≥ *ca.* −180 kJ mol^−1^). This relatively weak negative electrostatic potential implies that **1** would not be as strong a hydrogen-bond acceptor for water than **2**–**11**. However, molecules **2** and **3** do not form hydrates and are not outliers with negative potentials of −176 and −185 kJ mol^−1^, respectively.

### 3.5. Crystal packing analysis   

In order to address why **2** and **3** do not form hydrates as readily as **4**–**11**, we analyzed the crystal packing exhibited by **1**–**11**. There is a significant difference between the anhydrates and the hydrates. In the anhydrates, multiple weak C—H⋯π (edge-to-face, *D*_C⋯C_ = 3.53–3.79 Å) and/or C—H⋯N (*D*_C⋯N_ = 3.38–3.60 Å) interactions are responsible for controlling the crystal packing (Table S1). In the crystal structures of the hydrates, crystal packing tends to be directed by strong hydrogen bonds between water molecules (O—H⋯O with *D*_O⋯O_ = 2.74–2.86 Å) and between water molecules and basic nitrogen atoms (O—H⋯N with *D*_O⋯O_ = 2.82–2.98 Å). As mentioned earlier, for di- and trihydrates, in which one-dimensional water motifs are usually observed, water aggregation leads to the organization of organic molecules in such a manner that enables π–π stacking interactions (face-to-face, *D*_C⋯C_ = 3.44–3.91 Å). Thus, in the crystal structure of these hydrates, strong O—H⋯O and O—H⋯N hydrogen bonds are present along with π–π stacking interactions. The intermolecular interactions observed in the crystal structures of the hydrates and anhydrates are collected in Table S1.

**Figure 5:**
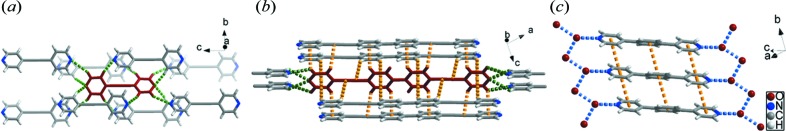
(*a*, *b*) Multiple C—H⋯N (green) and/or C—H⋯π (yellow) intermolecular interactions exist in the crystal structures of compounds **2** and **3**. (*c*) O—H⋯O and O—H⋯N hydrogen bonds (blue), and π–π stacking (face-to-face) interactions (yellow) are observed in the crystal structure of **10**·2H_2_O.

It has been reported that aromatic rings prefer to adopt edge-to-face or T-shaped geometry over face-to-face or parallel geometry (Hunter, 1994 ▸; Nishio, 2004 ▸). The crystal structures of both hydrate and anhydrate forms were determined for **4**–**7**. 7 × C—H⋯N (*D*_C⋯N_ = 3.38–3.60 Å) hydrogen bonds surround each molecule of **4** in its anhydrous form, whereas in the **4**·2H_2_O 4 × O—H⋯O (*D*_O⋯O_ = 2.74–2.75 Å), 2 × O—H⋯N (*D*_O⋯N_ = 2.83–2.87 Å), 3 × C—H⋯O (*D*_C⋯O_ = 3.40–3.55 Å), 4 × C—H⋯N (*D*_C⋯N_ = 3.38–3.60 Å) hydrogen bonds and 4 × π–π stacking interactions (face-to-face, *D*_C⋯C_ = 3.70–3.74 Å) are present. It is therefore unsurprising that **4** readily forms a dihydrate when subjected to our screening experiments. A comparison of intermolecular interactions in the anhydrous forms of **1**–**9** reveals that in **2**–**3**, for which hydrates do not yet exist, a greater number of C—H⋯N and/or C—H⋯π stacking interactions are observed. For example, there are more C—H⋯N (8 × *D*_C⋯N_ = 3.41 Å) and C—H⋯π (8 × *D*_C⋯C_ = 3.79 Å) interactions in **3** than in **4**–**6** (Fig. 5 ▸ *b*, Table S1). The propensity of **2** and **3** to exist as anhydrates might therefore be attributed to the large number of weak interactions they exhibit that would be lost in the corresponding hydrates.

## 4. Conclusion   

In this study we have investigated the propensity for hydrate formation of five- and six-membered *N*-heterocyclic aromatic compounds that are devoid of strong hydrogen-bond donors. Our investigation involving a CSD survey, systematic hydrate screening experiments, analyses of electrostatic potential maps and crystal packing patterns has led to the following conclusions:

CSD statistics tend to understate the propensity for hydrate formation when compared to systematic experimental screening that includes exposure to humidity and slurrying in water.

When hydrates are not afforded by systematic experimental screening, analysis of ESP maps and crystal packing in anhydrates can provide insight into why this is the case.

It would be inappropriate to extrapolate beyond the specific subset of molecular compounds studied herein, but systematic experimental studies on other subsets of molecular compounds are expected to provide insight into their propensity towards formation of crystalline hydrates.

## Supplementary Material

Crystal structure: contains datablock(s) 2, 3, 6, 6.2H2O, 7.4H2O, 9, 10.2H2O, 11.3H2O. DOI: 10.1107/S2052252516015633/lc5070sup1.cif

Structure factors: contains datablock(s) 2. DOI: 10.1107/S2052252516015633/lc50702sup2.fcf

Structure factors: contains datablock(s) 3. DOI: 10.1107/S2052252516015633/lc50703sup3.hkl

Structure factors: contains datablock(s) 6. DOI: 10.1107/S2052252516015633/lc50706sup4.hkl

Structure factors: contains datablock(s) 6.2H2O. DOI: 10.1107/S2052252516015633/lc50706.2H2Osup5.fcf

Structure factors: contains datablock(s) 7.4H2O. DOI: 10.1107/S2052252516015633/lc50707.4H2Osup6.hkl

Structure factors: contains datablock(s) 9. DOI: 10.1107/S2052252516015633/lc50709sup7.hkl

Structure factors: contains datablock(s) 10.2H2O. DOI: 10.1107/S2052252516015633/lc507010.2H2Osup8.fcf

Structure factors: contains datablock(s) 11.3H2O. DOI: 10.1107/S2052252516015633/lc507011.3H2Osup9.fcf

Synthetic procedures, PXRD and thermal analyses, detailed crystallographic information and additional figures. DOI: 10.1107/S2052252516015633/lc5070sup10.pdf

CCDC references: 1508120, 1509584, 1509585, 1509586, 1509587, 1509588, 1509589, 1509590

## Figures and Tables

**Table:** 

Compound	**2**	**3**	**6**	**6**·2H_2_O
Chemical formula	C_12_H_8_N_2_	C_26_H_16_N_2_	C_20_H_12_N_2_	C_20_H_16_N_2_O_2_
*M*_r_	180.20	356.41	280.32	316.35
*T* (K)	100 (2)	100 (2)	100 (2)	100 (2)
Crystal system	Orthorhombic	Monoclinic	Orthorhombic	Monoclinic
Space group	*Fddd*	*P*2_1_/*c*	*Pna*2_1_	*P*2_1_/*c*
*Z*	8	2	4	2
*a* (Å)	9.2584 (18)	22.1029 (5)	17.7436 (16)	14.957 (3)
*b* (Å)	12.936 (2)	5.62770 (10)	10.8510 (11)	4.8702 (9)
*c* (Å)	15.764 (3)	7.5548 (2)	7.5217 (7)	11.199 (2)
α (°)	90	90	90	90
β (°)	90	99.7070 (10)	90	103.719 (5)
γ (°)	90	90	90	90
*V* (Å^3^)	1888.0 (6)	926.28 (4)	1448.2 (2)	792.5 (3)
*D_x_* (Mg m^−3^)	1.268	1.285	1.286	1.326
μ (mm^−1^)	0.077	0.582	0.594	0.087
Measured/independent reflections (*R*_int_)	7024/664 (0.0772)	10 730/1625 (0.0179)	5947/2014 (0.1841)	10 327/1836 (0.1131)
Observed reflections [*I* > 2σ(*I*)]	478	1317	1112	1014
*R*_1_ †, *wR*_2_ ‡ [*I* > 2σ(*I*)]	0.0812, 0.2058	0.0464, 0.1436	0.0685, 0.1328	0.0784, 0.1254
*R*_1_, *wR*_2_ (all data)	0.1183, 0.2284	0.0548, 0.1519	0.1506, 0.1629	0.1676, 0.1498
Δρ_min_, Δρ_max_ (e Å^−3^)	−0.304, 0.390	−0.734, 0.184	−0.250, 0.241	−0.34, 0.274
Goodness-of-fit on *F* ^2^	1.144	1.098	1.011	1.070

**Table:** 

Compound	**7**·4H_2_O	**9**	**10**·2H_2_O	**11**·3H_2_O
Chemical formula	C_18_H_22_N_4_O_4_	C_20_H_20_N_2_	C_16_H_16_N_2_O_2_	C_24_H_24_N_18_O_5.5_
*M*_r_ (g mol^−1^)	358.39	288.38	268.31	652.61
*T* (K)	273 (2)	100 (2)	100 (2)	100 (2)
Crystal system	Triclinic	Orthorhombic	Monoclinic	Monoclinic
Space group	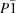	*Pna*2_1_	*P*2_1_/*c*	*C*2/*c*
*Z*	1	4	2	8
*a* (Å)	7.7923 (9)	21.105 (4)	7.4431 (4)	38.9915 (11)
*b* (Å)	7.9651 (9)	6.5848 (11)	3.9111 (2)	6.9971 (2)
*c* (Å)	8.4455 (10)	11.241 (2)	22.7679 (12)	29.1584 (9)
α (°)	102.627 (3)	90	90	90
β (°)	95.961 (3)	90	99.239 (4)	130.424 (2)
γ (°)	114.174 (3)	90	90	90
*V* (Å^3^)	455.51 (9)	1562.2 (5)	654.19 (6)	6056.0 (3)
ρ_calc_ (g cm^−3^)	1.307	1.226	1.362	1.432
μ (mm^−1^)	0.094	0.072	0.735	0.919
Measured/independent reflections (*R*_int_)	6294/2134 (0.0422)	37 588/3625 (0.1743)	6041/1261 (0.1050)	37 075/5171 (0.0859)
Observed reflections [*I* > 2σ(*I*)]	1325	2466	876	4014
*R*_1_ †, *wR*_2_ ‡ [*I* > 2σ(*I*)]	0.1018, 0.1549	0.0745, 0.1295	0.0888, 0.1820	0.0536, 0.1288
*R*_1_, *wR*_2_ (all data)	0.1665, 0.1736	0.1286, 0.1467	0.1322, 0.2074	0.0744, 0.1407
Δρ_min_, Δρ_max_ (e Å^−3^)	−0.348, 0.217	−0.280, 0.456	−0.439, 0.652	−0.555, 0.991
Goodness-of-fit on *F* ^2^	1.134	1.065	1.066	1.036

† *R*_1_ = ∑||*F*_o_| − |*F*_c_||/∑|*F*_o_|.

‡ *wR*_2_ = {∑[*w*(*F*_o_^2^∑*F*_c_^2^)^2^]/∑[*w*(*F*_o_^2^)]}^1/2^.

**Table 2:** Results of hydrate screening experiments that afforded anhydrous (A) and/or hydrated (H) forms

Compound	Slurry in H_2_O	75% R.H./40°C	Competitive slurry	SDG†	Hydrate stability in air
**1**	A	A	–	A	–
**2**	A	A	–	A	–
**3**	A	A	–	A	–
**4**	H	H	H	H	> 30 d
**5**	H	H	H	A + H	10 d < H < 30 d
**6**	H	H	H	A + H	> 30 d
**7**	H	H	H	A + H	< 1 d
**8**	H	H	H	A	< 1 d
**9** ‡	H	H	H	–	–
**10**	H	H	H	A + H	< 1 d
**11**	H	H	H	A + H	> 30 d

† For compounds **1**–**3**, SDG was performed for 30 min each.

‡ Experiments to determine the stability of the hydrate of **9** were not performed due to the similarity of the powder patterns of the hydrated and the anhydrous forms.
