# Supplementary material for: Towards an understanding of the propensity for crystalline hydrate formation by molecular compounds
Source: IUCrJ. 2016 Oct 18;3(Pt 6):430–9. doi: 10.1107/S2052252516015633 (PMC5094445; doi:10.1107/S2052252516015633)
Supplement: Supplementary file 10 [file m-03-00430-sup10.pdf]

IUCrJ

Volume 4 (2017)

Supporting information for article:

Towards an understanding of the propensity for crystalline hydrate formation by molecular compounds

Alankriti Bajpai, Hayley S. Scott, Tony Pham, Kai-Jie Chen, Brian Space, Matteo Lusi, Miranda L. Perry and Michael J. Zaworotko

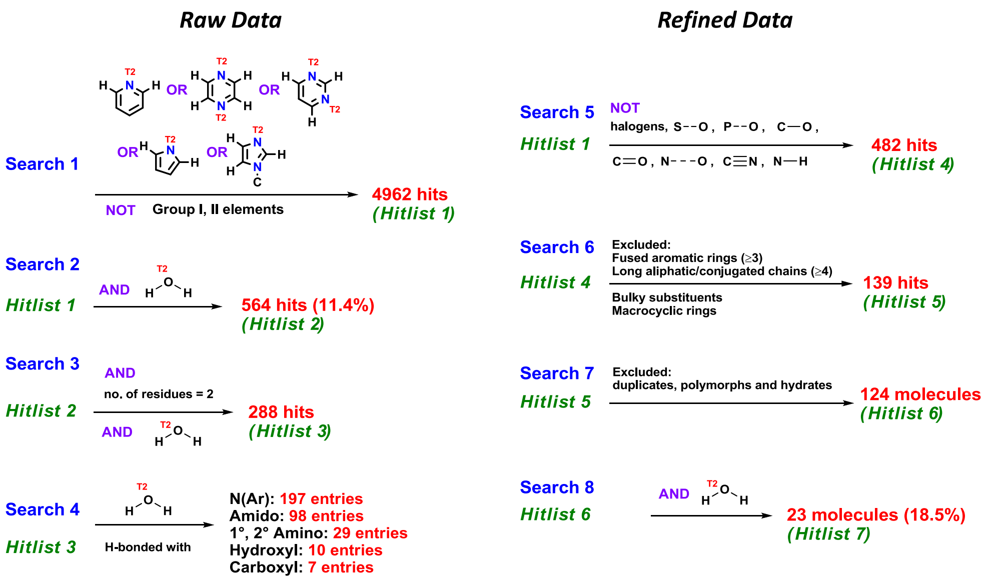

Figure S1 Summary of CSD searches conducted herein.

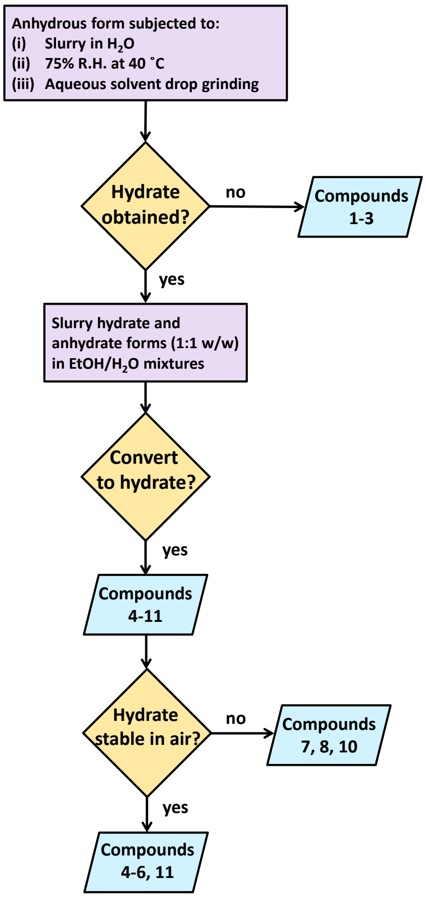

Figure S2 Breakdown of the flow of experimental work.

S1. SYNTHESIS AND CHARACTERIZATION OF COMPOUNDS

S1.1. General procedure for Sonogashira cross-coupling reactions

Compounds **2**, **3** and **6** were prepared by Pd^0-catalysed Sonogashira coupling of 4-ethynylpyridyine hydrochloride with the corresponding mono- or diiodo derivatives.

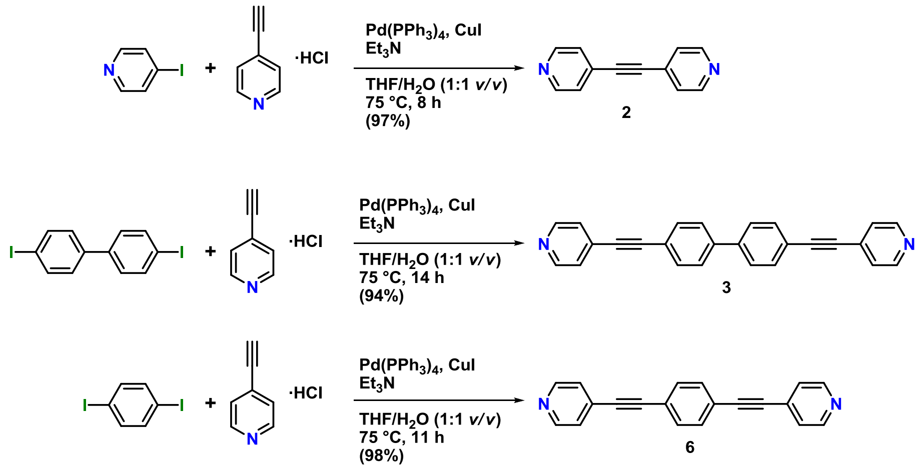

1,2-Bis(4-ethynylpyridyl)acetylene (2). In oven-dried 250 mL two-neck round bottom flask equipped with a reflux condenser and N_2 supply, 4-ethynylpyridyine hydrochloride (3.67 g, 17.90 mmol) and Et_3N (40.0 mL, 2 mL/mmol of 4-iodopyridine) were dissolved in 100 mL of THF/H_2O (1:1 v/v) mixture. The contents were allowed to stir at rt for 10 min followed by addition of 4-iodopyridine (3.0 g, 21.49 mmol), CuI (0.34 g, 1.80 mmol) and $Pd(PPh_3)_4$ (1.0 g, 0.90 mmol). After this, the reaction mixture was heated at reflux for 8 h. The progress of reaction was monitored by thin layer chromatography (TLC). At the end of the reaction, the reaction mixture was dried *in vacuo*, solid residue extracted using dichloromethane (DCM). The combined DCM extract was washed with brine solution, dried over anhyd Na_2SO_4 and concentrated *in vacuo*. Silica gel column chromatography was performed using 40% $CHCl_3$/pet. ether as eluent to obtain pure product as a pale yellow solid; yield 3.75 g, 97%; mp 161–165 °C; ^{1}H NMR (270 MHz, $CDCl_3$) δ 7.42 (4H, d, J = 5.80 Hz), 8.66 (4H, d, J = 5.80); ^{13}C NMR (67.5 MHz, $CDCl_3$) δ 93.2, 125.5, 131.9, 149.8.

A similar procedure as described above was followed to prepare compounds **3** and **6** using 0.5 equiv of corresponding diiodoarene (4,4'-diiodobiphenyl and 1,4-diiodobenzene, respectively) with respect to all other reactants and reagents.

4,4'-Bis(4-ethynylpyridyl)biphenyl (3): light brown solid; 94% yield; ^{1}H NMR (270 MHz, $CDCl_3$) δ 7.38 (4H, d, J = 4.55 Hz), 7.63 (8H, s), 8.60 (4H, d, J = 4.55 Hz); ^{13}C NMR (67.5 MHz, $CDCl_3$) δ 87.7, 93.6, 121.6, 125.6, 131.3, 132.4, 140.7, 149.8.

1,4-Bis(4-pyridyl)benzene (6): yellow solid; 98% yield; ^{1}H NMR (270 MHz, $CDCl_3$) δ 7.38 (4H, d, *J* = 6.18 Hz), 7.56 (4H, s), 8.61 (4H, d, *J* = 6.18 Hz); ^{13}C NMR (67.5 MHz, $CDCl_3$) δ 89.0, 93.6, 123.1, 125.5, 131.7, 132.6, 159.0.

S1.2. General procedure for Suzuki cross-coupling reaction

Compounds **9** and **10** were synthesised by 2-fold Pd^0-catalysed Suzuki coupling of 4-pyridynylboronic acid with 1,4-dibromobenzene and 1,4-dibromodurene, respectively.

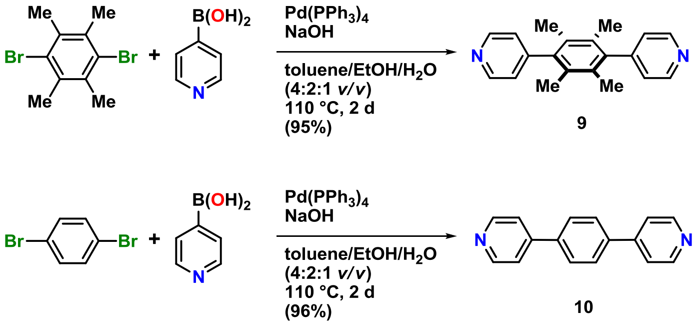

1,4-Bis(4-pyridyl)durene (9). A 250 mL oven-dried two-necked round bottom flask was cooled under N_2 atmosphere and charged with 1,4-dibromodurene (2.0 g, 6.85 mmol), 4-pyridinylboronic acid (2.53 g, 20.5 mmol), $Pd(PPh_3)_4$ (0.39 g, 0.42 mmol), powdered NaOH (1.10 g, 27.4 mmol), 30 mL of toluene, 20 mL of EtOH and 10 mL of distilled water. The resultant reaction mixture was refluxed at 110 °C. The contents dissolved completely to give clear yellow coloration over a period of 1.5 h. Heating was continued at reflux under N_2 atmosphere for 2 d. The change in the color of reaction mixture from yellow to dark brown indicated completion of the reaction, which was further verified by TLC. Subsequently, the reaction mixture was cooled and extracted with $CHCl_3$ and washed with brine solution. The organic phase was dried over anhyd Na_2SO_4 and concentrated *in vacuo*. The pure product was isolated by Silica gel column chromatography using $CHCl_3$/pet. ether (40%) mixture as an eluent to afford the product **9** as a white solid in 95% yield (1.45 g, 3.02 mmol); ^{1}H NMR ($CDCl_3$, 270 MHz) δ 1.92 (12H, s), 7.12 (4H, d, *J* = 5.31 Hz), 8.69 (4H, d, *J* = 5.31 Hz); ^{13}C NMR (67.5 MHz, $CDCl_3$) δ 17.9, 124.7, 131.3, 139.3, 150.0.

1,4-Bis(4-pyridyl)benzene (10). A similar procedure as described for the preparation of **9** was followed, which involves 2-fold Pd^0-catalyzed Suzuki coupling of 1,4-dibromobenzene with 4-pyridinylboronic acid leading to 1,4-bis(4-pyridyl)benzene (**10**); colorless solid; 96% yield; ^{1}H NMR ($CDCl_3$, 270 MHz) δ 7.55 (4H, d, *J* = 5.44 Hz), 7.76 (4H, s), 8.68 (4H, d, *J* = 5.44 Hz); ^{13}C NMR (67.5 MHz, $CDCl_3$) δ 120.7, 137.6, 147.1, 149.3.

S2. CRYSTAL PACKING DESCRIPTIONS

1,2-bis(4-pyridyl)acetylene (2). Compound **2** crystallises in the orthorhombic space group *Fddd* with a quarter of molecule **2** comprising the asymmetric unit. The torsion angle between the two pyridyl rings is 48.1˚. The crystal structure was found to contain multiple weak C–H···N hydrogen bonds ($d_{A\text{–}H\cdots B}$ = 2.47 Å, $D_{A\cdots B}$ = 3.38 Å, $\angle_{A\text{–}H\cdots B}$ = 158.6˚, Table S1) as shown in Fig. S3c.

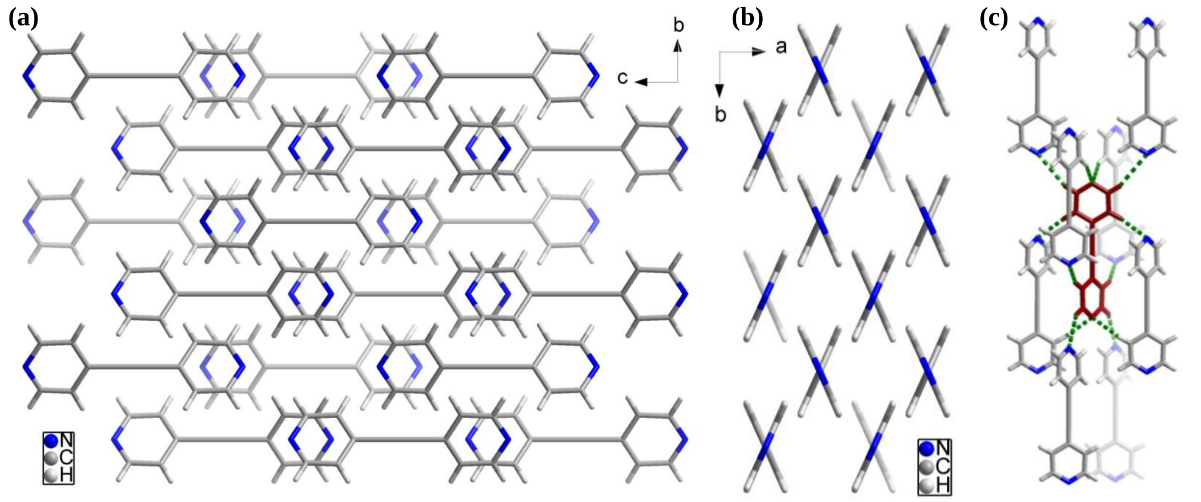

Figure S3 (a,b) Crystal packing depicted down *a*- and *c*-axes. (c) C–H···N interactions between the adjacent molecules.

4,4'-bis(4-ethynylpyridyl)biphenyl (3). Compound **3** crystallises in the monoclinic space group $P2_1/c$ with half a molecule comprising the asymmetric unit. Each molecule shows multiple C–H···N and C–H···$\pi_{acetylenyl}$ interactions with neighbouring molecules (Fig. S4c).

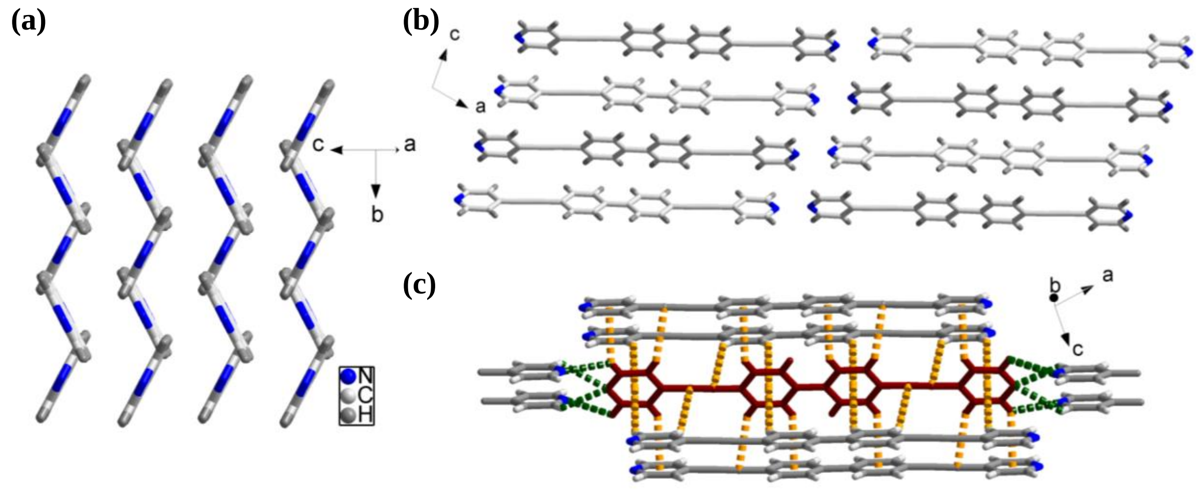

Figure S4 (a,b) Crystal packing diagrams of anhydrous form of **3** showing the arrangement of molecules. (c) The multiple C–H···N (green) and C–H···π (yellow) interactions around each molecule (red).

1,4-bis(4-ethynylpyridyl)benzene (6). Compound **6** crystallises in the orthorhombic space group $Pna2_1$ with one molecule of **6** comprising the asymmetric unit. The crystal structure was found to contain multiple weak C–H···N and C–H···π interactions (Fig. S5b, Table S1).

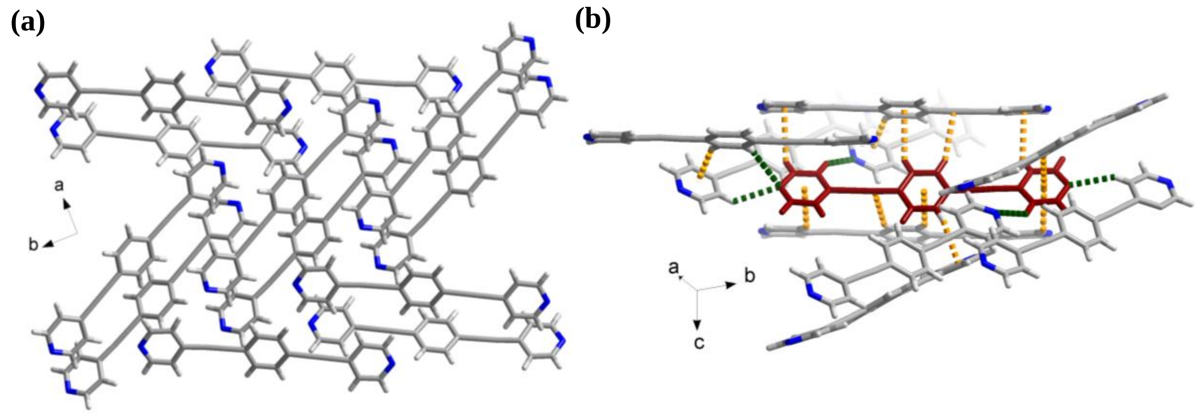

Figure S5 (a) Crystal packing of anhydrous form of **6** depicted down *c*-axis. (b) Multiple weak C–H···N (green) and C–H···π (yellow) interactions around each molecule (red) are shown.

1,4-bis(4-ethynylpyridyl)benzene (6·2H$_2$O). Compound **6**·2H_2O crystallises in the monoclinic space group $P2_1/c$ with half a molecule of **6** and one molecule of water comprising the asymmetric unit. Each water molecule serves as a two H-bond donor for one pyridyl group and water molecule, and one H-bond acceptor for a water molecule. The water molecules form 1D zigzag (C2) chains via O–H···O hydrogen bonds, which run along *b*-axis (Fig. S6a). The aromatic nitrogen atoms interlink these C2 chains via O–H···N hydrogen bonding with the water molecules leading to formation of 2D layers.

These 2D layers are found to stack along the *c*-axis as shown in Fig. S6b. The bond distances and angles for these intermolecular interactions are given in Table S1.

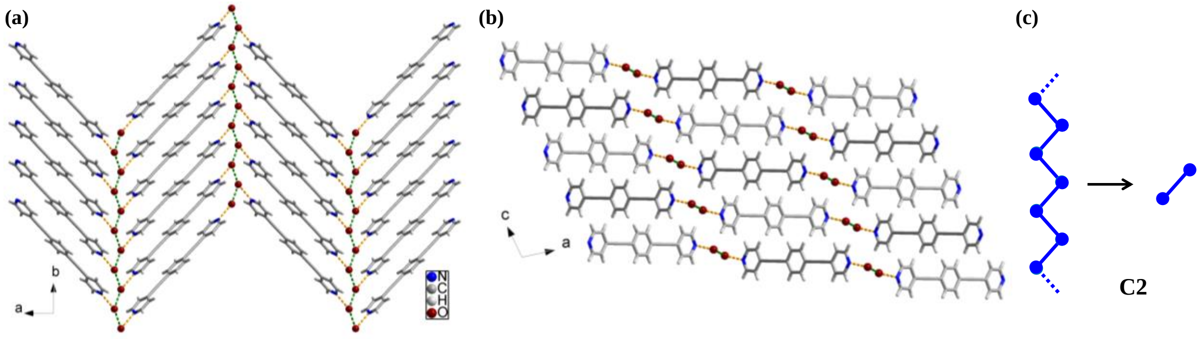

Figure S6 (a) 2D layers formed by the association of molecules of **6** with the 1D infinite zigzag chains of water. (b) The crystal packing diagram showing the stacking of 2D layers. (c) The schematic of C2 water cluster is shown.

Bis(pyridin-4-ylmethylene)benzene-1,4-diamine tetrahydrate (7·4H_2O). Compound **7**·4H_2O crystallises in the triclinic *P-1* space group with half a molecule of **7** and two molecules of water comprising the asymmetric unit. A closer inspection of the crystal structure shows that the water molecules form a discrete tetrameric (R4) cluster via O-H···O H-bonding. For each tetramer, two water molecules are hydrogen bonded to two pyridyl nitrogens of **7**, while the other two water molecules hydrogen bond to the imino nitrogen atoms (Fig. S7a). As a result, 2D layers are formed (Fig. 7a). These layers interact with the adjacent layers through multiple C-H···O H-bonds (Table S1) thereby giving rise to an overall 3D structure, Fig. 7b.

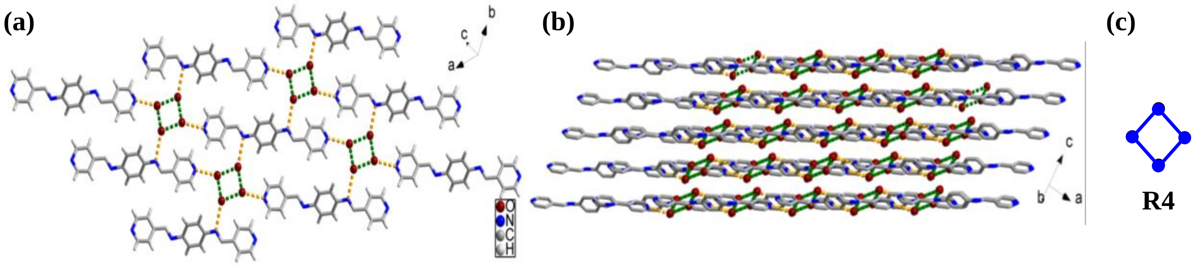

Figure S7 (a) 2D layers formed by O-H···O H-bonding between molecules of **7** and tetrameric water clusters. (b) The crystal packing diagram showing the stacking of 2D layers. (c) The schematic of R4 water cluster is shown.

1,4-Bis(4-pyridyl)durene 9. Compound 9 crystallises in the orthorhombic space group $Pna2_1$ with one molecule of **9** comprising the asymmetric unit. The two pyridyl rings are found to subtend torsion angles of 81.7 and 89.6˚ with respect to the central durenyl core. Each molecule of **9** (depicted red in Fig. S8b) is found to exhibit multiple C–H···N and C–H···π interactions with its neighbouring molecules. The various bond distances and angles of these interactions are given in Table S1.

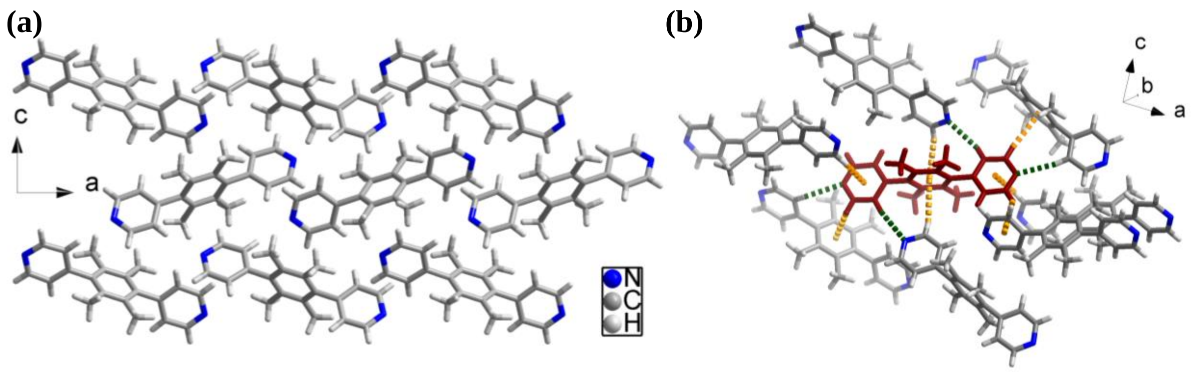

Figure S8 (a) Crystal packing diagram of **9**. (b) All the C–H···N (green) and C–H···π interactions (yellow) around one of the molecules of **9** (red) are shown.

1,4-Bis(4-pyridyl)benzene dihydrate (10·2H$_2$O). Compound **10**·2H$_2$O crystallises in the monoclinic space group $P2_1/c$ with half a molecule of **10** and one water molecule comprising the asymmetric unit. Each water molecule acts as a two H-bond donor for one pyridyl group and water molecule, and one H-bond acceptor for a water molecule. The water molecules thus form 1D zigzag (C2) chains, which run along *b*-axis. These 1D chains are interlinked by molecules of **10** to form 2D sheets down *b*-axis. Molecules of **10** belonging to one layer are found to display π-π stacking interactions with those of the 2D layers on either side (Table S1).

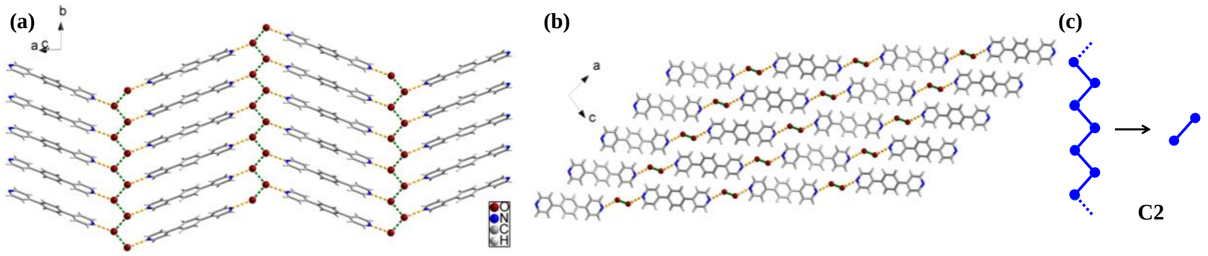

Figure S9 (a) A 2D layer showing the 1D (C2) chains of water molecules that are interlinked by molecules of **10**. (b) The crystal packing diagram showing the stacking of these 2D layers. (c) The schematic of C2 water cluster is shown.

2,4,6-Tris(imidazol-1-yl)-1,3,5-s-triazine (11·3H$_2$O). Compound **11**·3H$_2$O crystallises in the monoclinic space group $C2/c$ with two molecules of **11** and six water molecules comprising the asymmetric unit. Two different water clusters (1D tapes) exist as pentagonal (T5(2)) and hexagonal (T6(1)) rings, Fig. S10b. Two nitrogen atoms of every molecule of **11** form O–H···N hydrogen bonds with two independent water molecules ($D_{O\cdots O}$ = 2.82 to 2.90 Å). The water tapes interlink the molecules of **11**, which are in turn re-enforced by π-π stacking (face-to-face) interactions, Fig. S10. Water molecules in these tapes are O–H···O hydrogen bonded (2.78–2.85 Å for pentagon and 2.95–3.04 Å for hexagon).

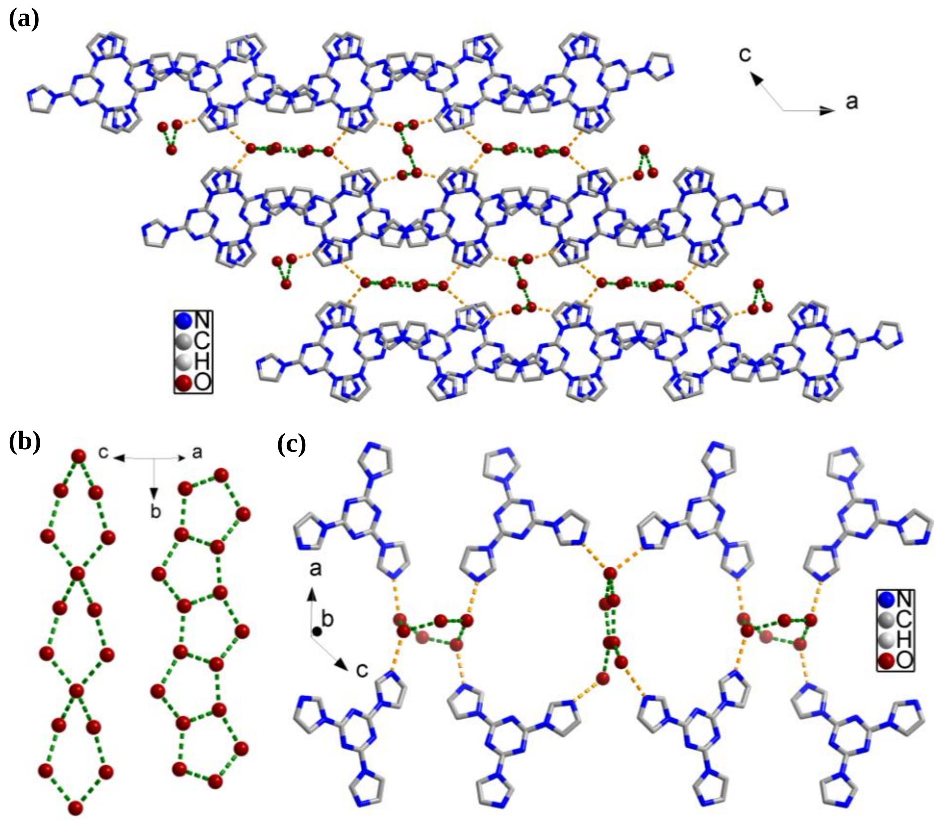

Figure S10 (a) Crystal packing diagram of **11**·3H_2O. (b) Water molecules assemble into pentameric (T5(2)) and hexameric (T6(1)) 1D infinite tapes, which propagate along *b*-axis. (c) The molecules of **11** are interlinked by these 1D water clusters.

Table S1. The intermolecular interactions around each molecule in the crystal structures of compounds **1-11**.

Compound	Total number of intermolecular interactions	Type of intermolecular interaction	$d_{A–H\cdots B}$ (Å)	$D_{A\cdots B}$ (Å)	$\angle_{A–H\cdots B}$ (°)
**1**	8	C–H···N	2.63	3.49	153.1
**2**	8	C–H···N	2.47	3.38	158.6
**3**	8	C–H···N	2.73	3.41	130.1
	8	C–H···$\pi_{acetylenyl}$	2.98	3.79	143.4
**4**	7	C–H···N	2.63	3.57	156.5
			2.61	3.51	173.3
			2.74	3.60	145.9
			2.63	3.50	154.9
			2.48	3.44	170.9
**5**	4	C–H···N	2.53	3.48	157.2
	2	π-π stacking (face-to-face)	-	3.91	-
**6**	2	C–H···N	2.73	3.57	147.6
	2	C–H··· $\pi_{acetylenyl}$	3.02	3.90	153.9
**7**	6	C–H···N	2.70	3.49	144.2
			2.56	3.44	159.0
	3	C–H··· π_{Ar} (edge-to-face)	2.65	3.53	159.0
	1	π-π stacking (face-to-face)	-	3.65	-
**8**	4	C–H··· π_{Ar} (edge-to-face)	2.98	3.70	129.5
**9**	4	C–H···N	2.66	3.45	140.7
			2.70	3.44	134.6
			2.65	3.59	170.1
	4	C–H··· π_{Ar} (edge-to-face)	2.87	3.79	164.2
			2.95	3.53	121.0
**4**·$2H_2O$	2	O–H···N	1.99	2.87	164.3
			1.99	2.84	154.9
			1.99	2.83	154.5
			2.01	2.87	161.5
	4	O–H···O	1.84	2.74	176.2
			1.85	2.75	174.2
	4	C–H···N	2.67	3.57	159.7
			2.69	3.60	159.7
	3	C–H···O	2.46	3.40	169.9
			2.60	3.55	171.0
	4	π_{Ar}-π_{Ar} stacking (face-to-face)	-	3.70	-
			-	3.74	-
**5**·H_2O	2	O–H···N	2.04	2.88	159.0
			2.08	2.90	160.2
	2	C–H···N	2.74	3.59	137.7
	2	C–H···O	2.39	3.28	157.2
			2.62	3.38	136.9
	2	C–H··· π_{Ar} (edge-to-face)	3.42	3.82	108.1
**6**·$2H_2O$	2	O–H···N	1.88	2.85	157.9
	3	O–H···O	1.96	2.82	166.8
	2	C–H···O	2.44	3.38	169.9
	4	π_{Ar}-$\pi_{acetylenyl}$ stacking	-	3.44	-
**7**·H_2O	2	O–H···N	2.20	3.01	158.4
	1	C–H···O	2.48	3.31	148.0
	2	C–H···N	2.70	3.56	153.5

	4	O–H···N	2.07	2.87	163.9
			2.24	2.98	168.1
**7**·4H_2O	4	C–H···O	2.72	3.58	155.1
			2.24	2.98	168.1
	4	O–H···O	2.11	2.84	153.5
			1.98	2.80	167.0
**10**·2H_2O	2	O–H···N	1.83	2.85	160.9
			2.63	3.39	137.7
	6	C–H···O	2.71	3.65	170.3
			2.83	3.24	106.7
	3	O–H···O	1.70	2.71	174.8
	6	π_{Ar}-π_{Ar} stacking (face-to-face)	-	3.91	-
**11**·3H_2O	2	O–H···N	2.07	2.91	166.5
			1.90	2.85	167.4
			-	2.84	-
			-	2.82	-
	12	O–H···O	1.95	2.86	172.8
			1.93	2.84	169.2
	8	π_{Ar}-π_{Ar} stacking (face-to-face)	-	3.58	-
			-	3.48	-
			-	3.81	-

Note: The centroids of the π-systems were created in order to measure the bond distances and angles of the corresponding interactions.

S3. RESULTS OF HYDRATE SCREENING EXPERIMENTS

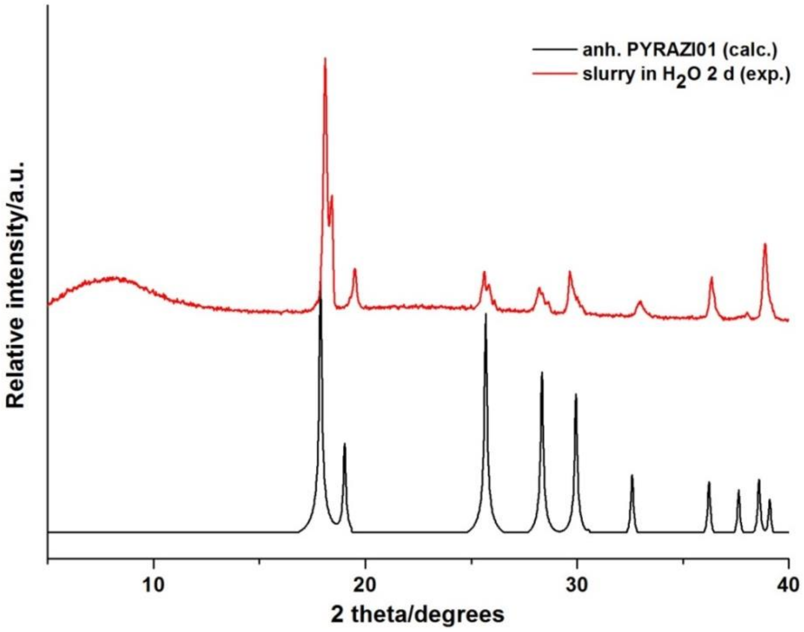

Figure S11 PXRD pattern for **1** after slurrying in water at rt for 2 d.

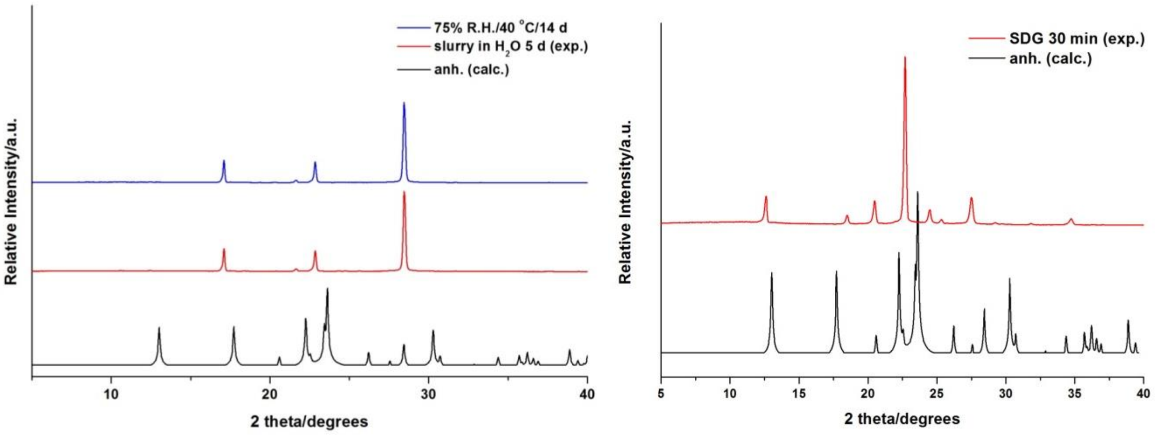

Figure S12 PXRD patterns for **2** after slurrying in water and humidity experiments.

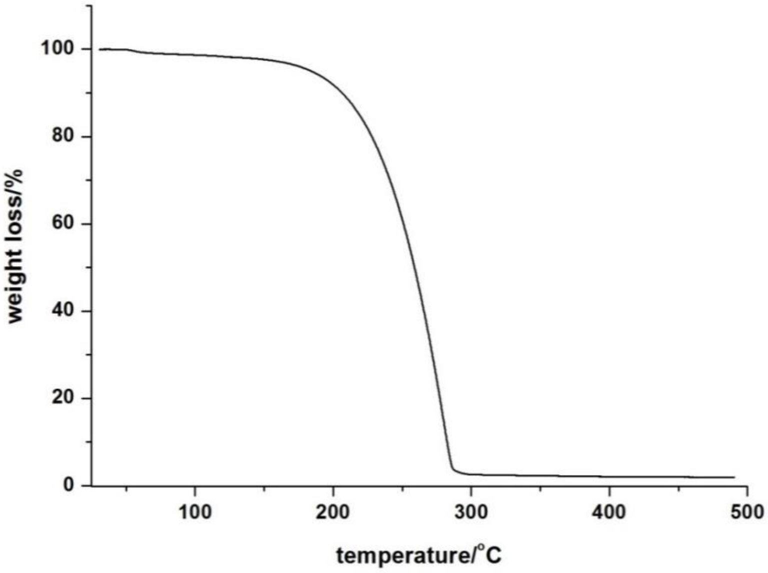

Figure S13 TGA profile for **2** after slurrying in water at rt for 5 d.

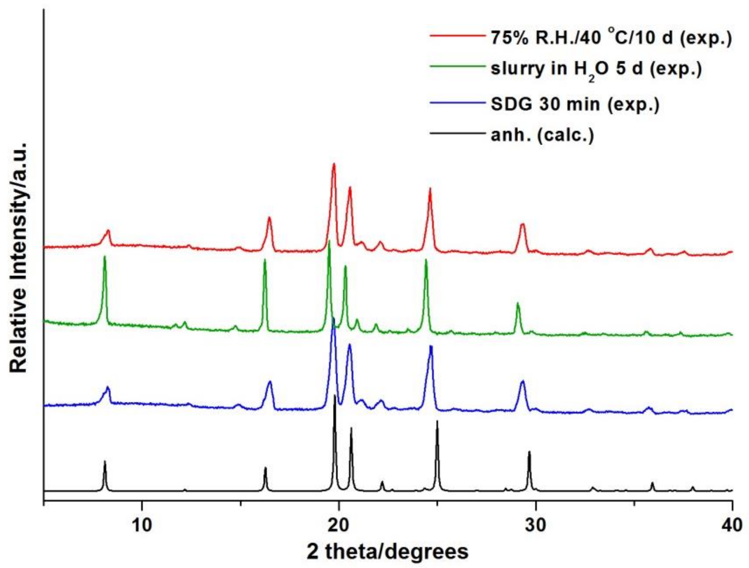

Figure S14 PXRD patterns for **3** isolated after SDG, slurrying in water and exposure to humidity.

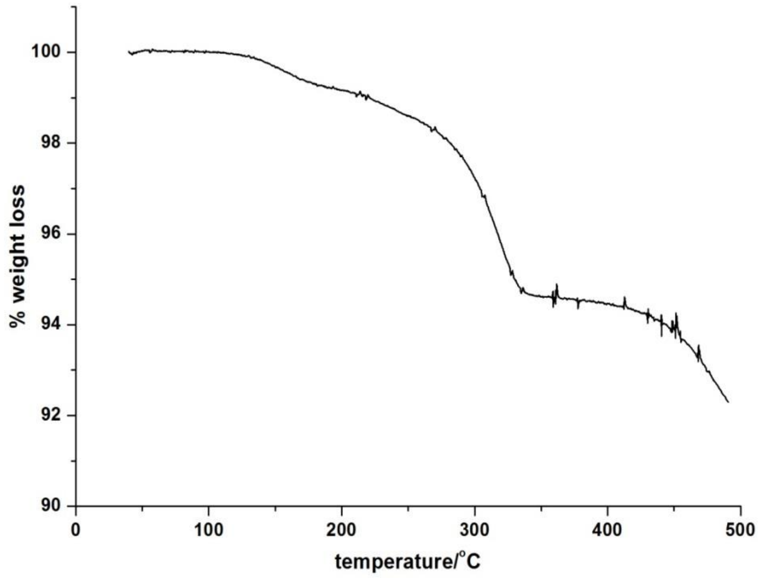

Figure S15 TGA profile of **3** after slurry in water at rt for 5 d.

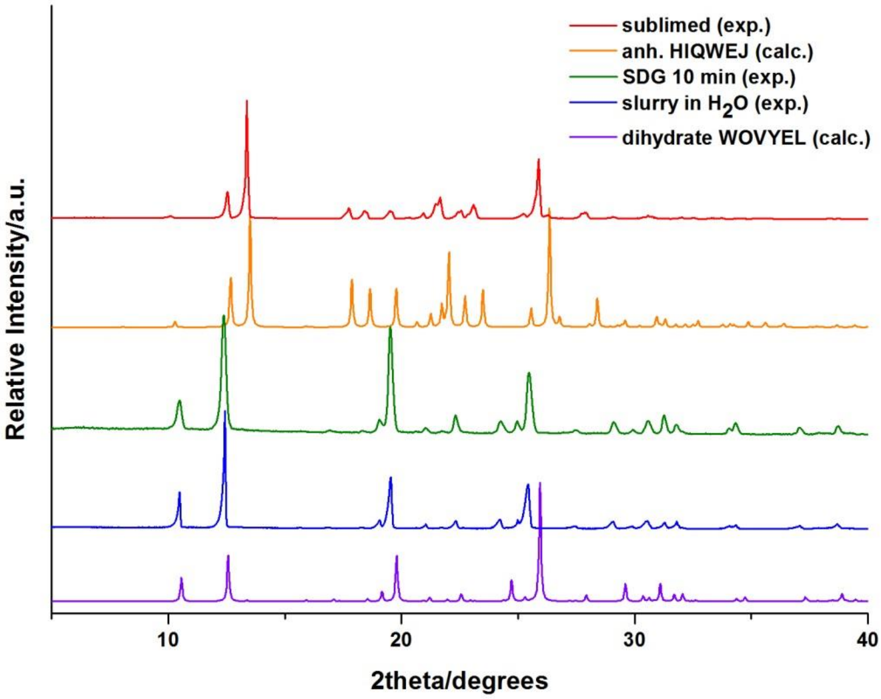

Figure S16 PXRD patterns for anhydrous and hydrated forms of **4**. The dihydrate (**4**·$2H_2O$) was isolated from water slurry in 1 d and solvent-drop grinding (SDG) using water in 10 min.

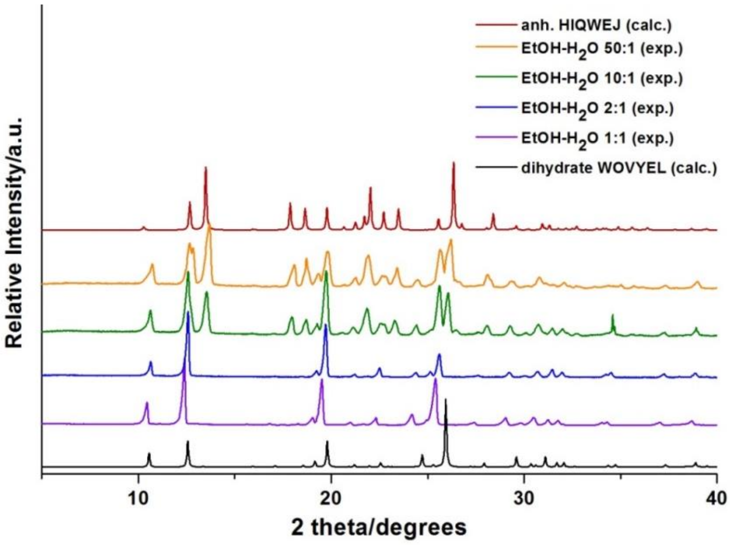

Figure S17 PXRD patterns for competitive slurry experiments for 1:1 w/w mixture of anhydrous and hydrated forms of **4** at rt for 2 d.

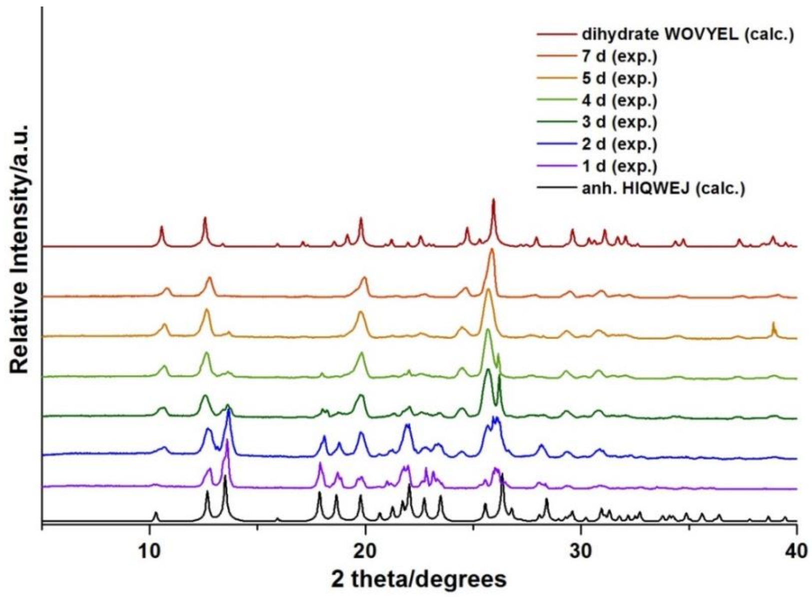

Figure S18 PXRD patterns showing gradual conversion of anhydrous form of **4** into hydrated form upon exposure to 75% R.H. at 40 ˚C.

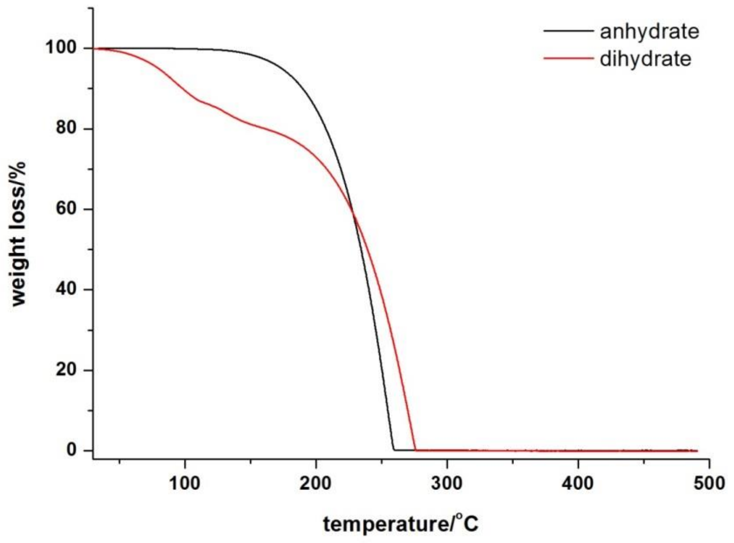

Figure S19 TGA profiles for the anhydrous and hydrated forms of **4.**

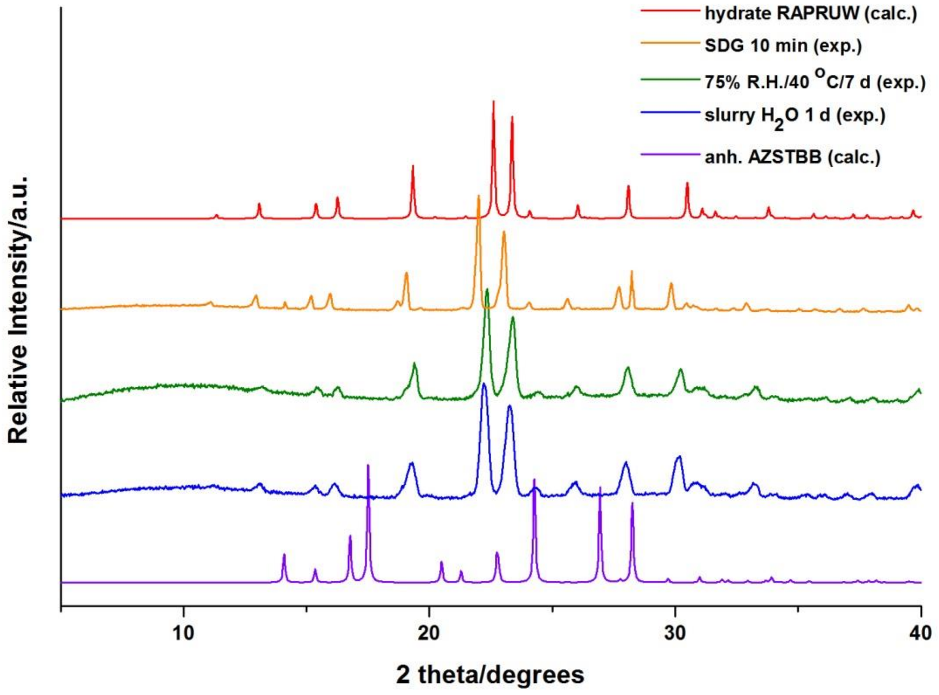

Figure S20 PXRD patterns for hydrated and anhydrous forms of **5**.

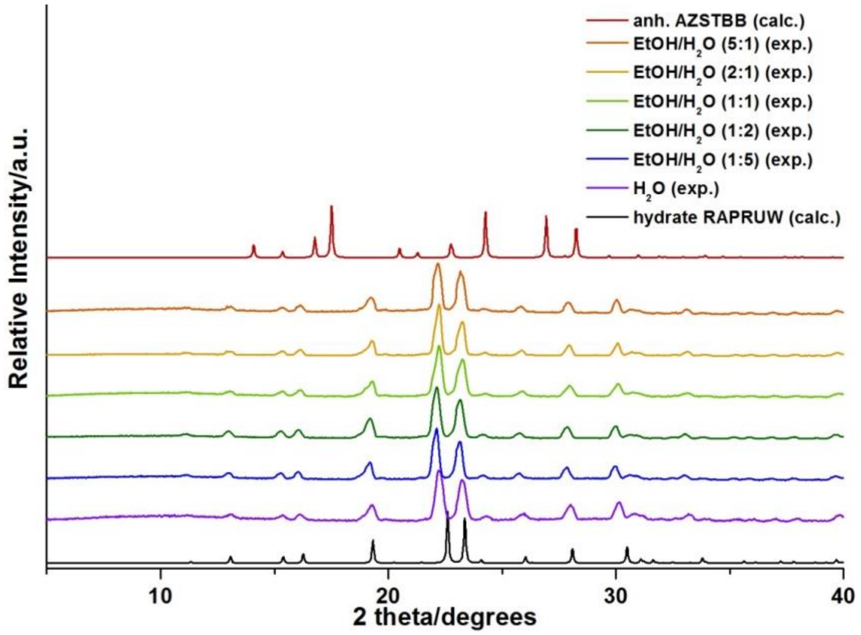

Figure S21 PXRD patterns for competitive slurry experiments for 1:1 w/w mixture of anhydrous and hydrated forms of **5** at rt for 2 d.

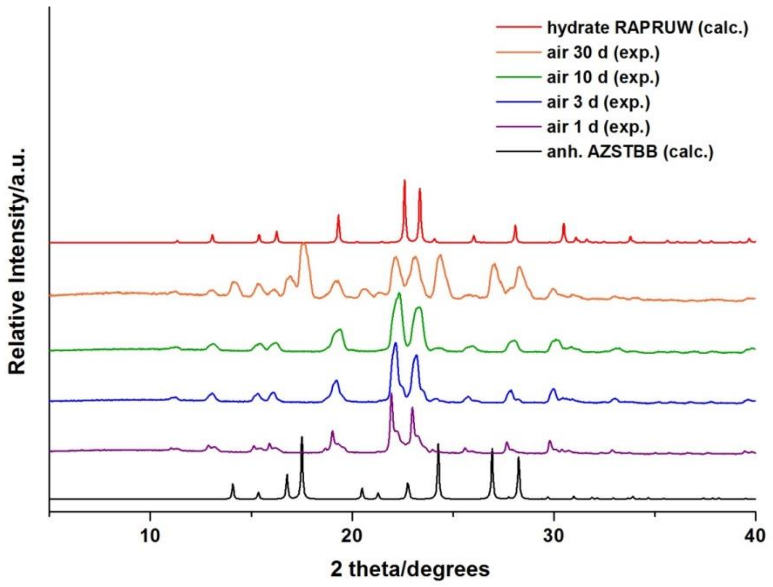

Figure S22 PXRD patterns showing the stability of hydrate form of **5** in air.

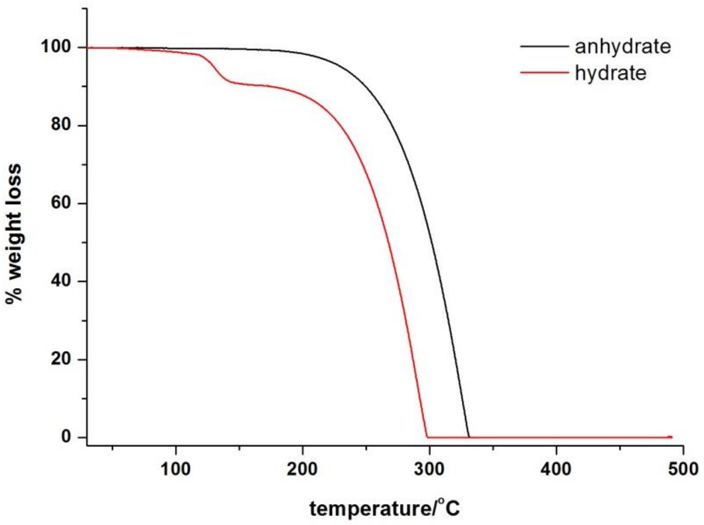

Figure S23 TGA profiles for the anhydrous and hydrated forms of **5**.

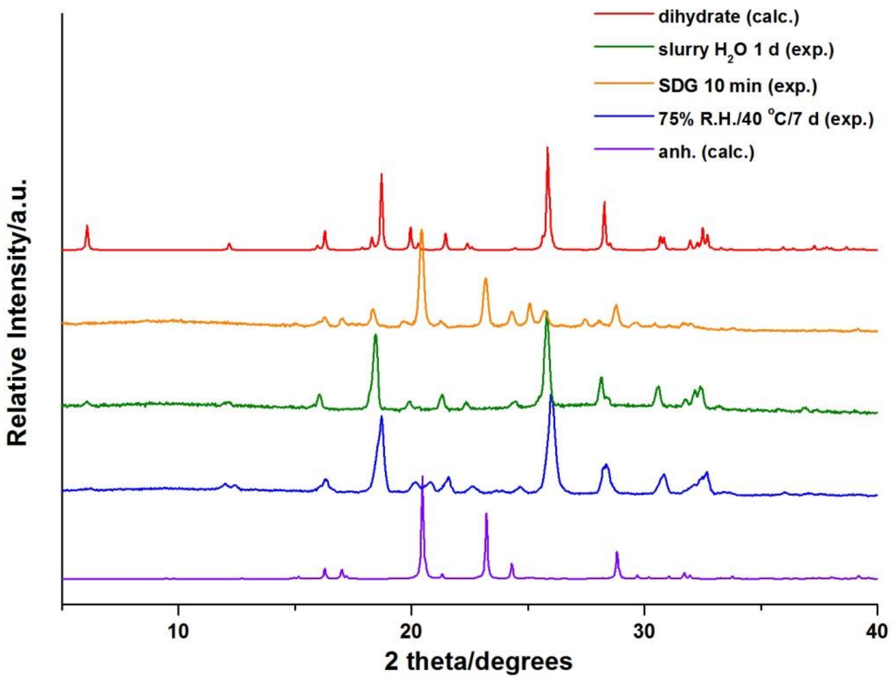

Figure S24 PXRD patterns for anhydrous and hydrated (**6**·2H_2O) forms of **6**.

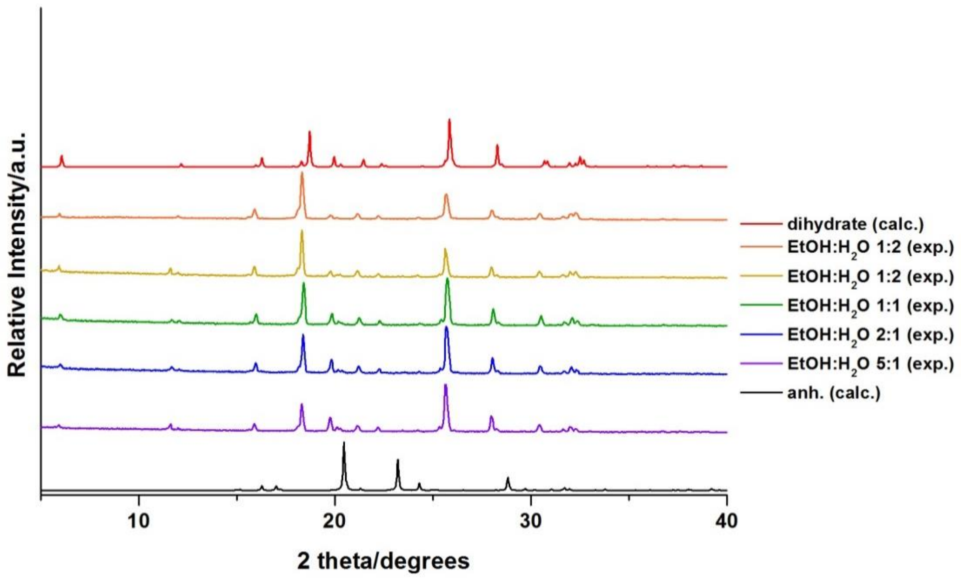

Figure S25 PXRD patterns for competitive slurry experiments for 1:1 w/w mixture of anhydrous and hydrated (**6**·2H_2O) forms of **6** at rt for 2 d.

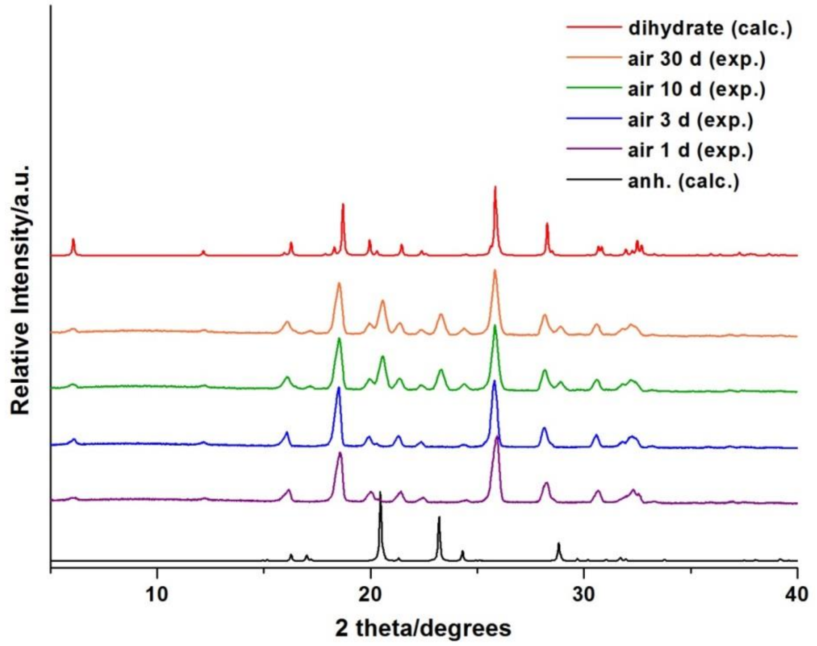

Figure S26 PXRD patterns showing the stability of hydrate form of 6 in air.

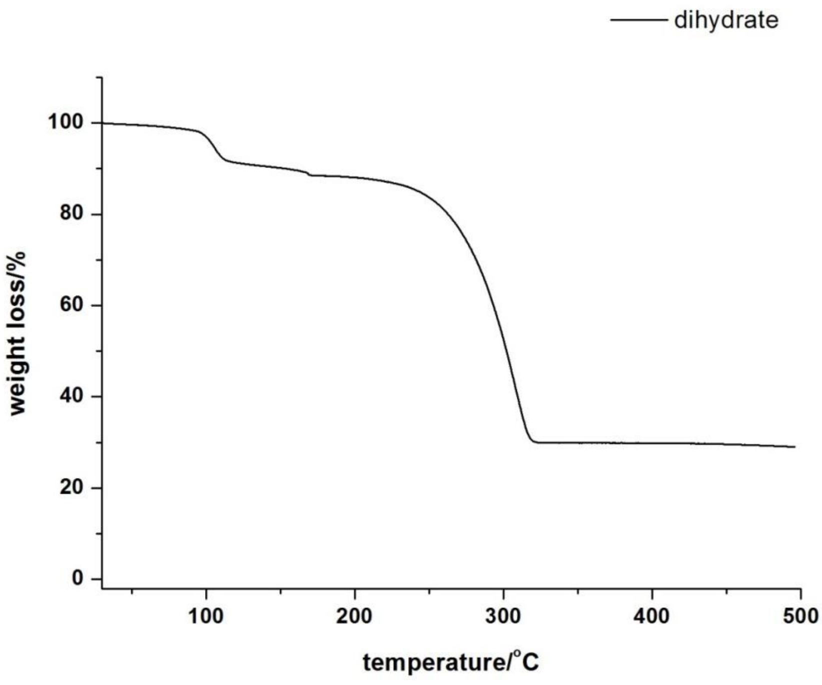

Figure S27 TGA profile for hydrate of **6** (**6**·$2H_2O$).

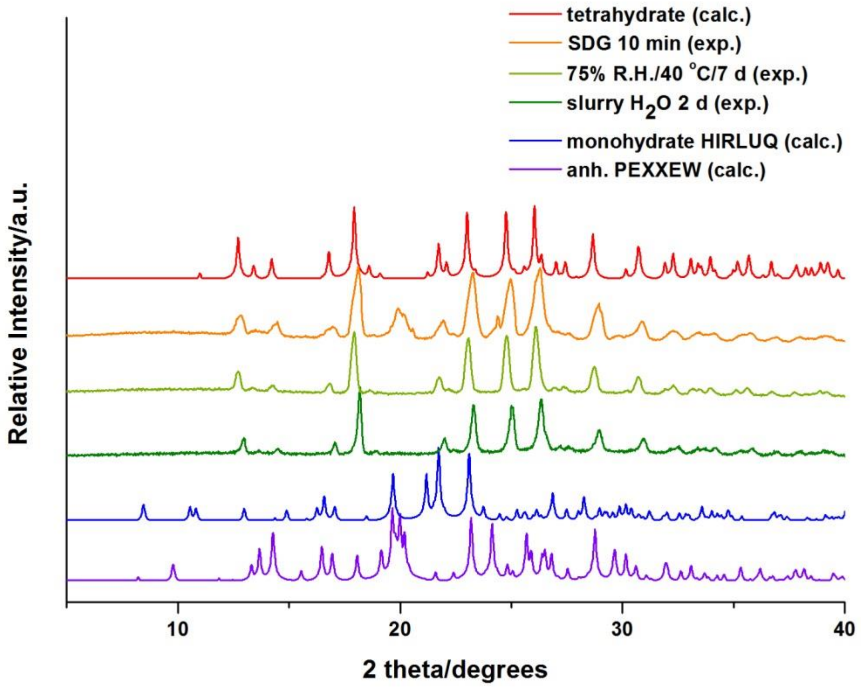

Figure S28 PXRD patterns for anhydrous and hydrated forms of **7**.

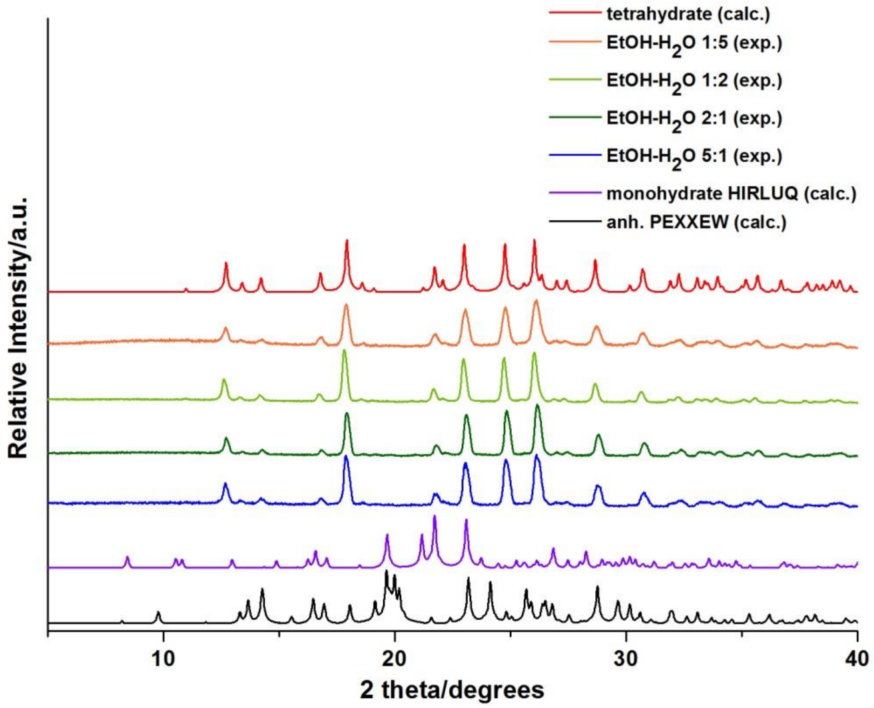

Figure S29 PXRD patterns for competitive slurry experiments for 1:1 w/w mixture of anhydrous and hydrated forms of **7** at rt for 2 d.

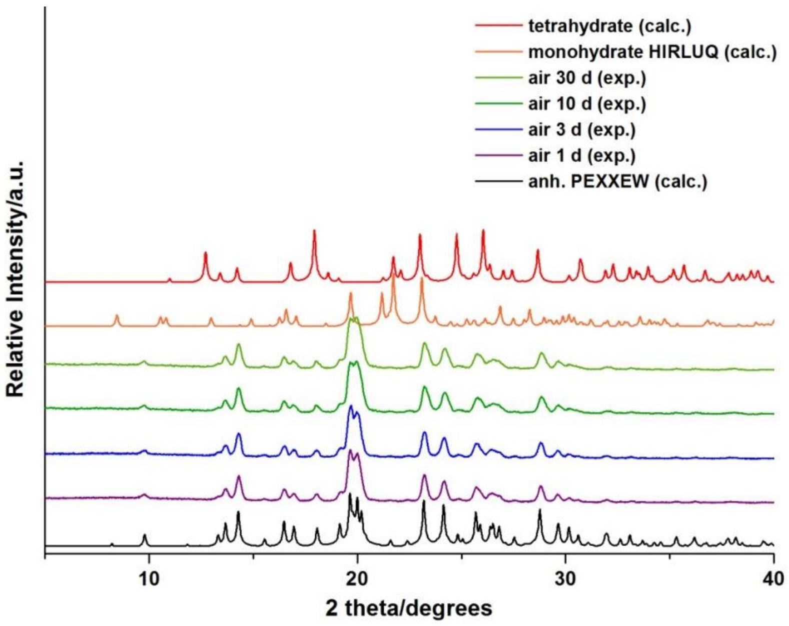

Figure S30 PXRD patterns showing the stability of hydrate form of **7** in air.

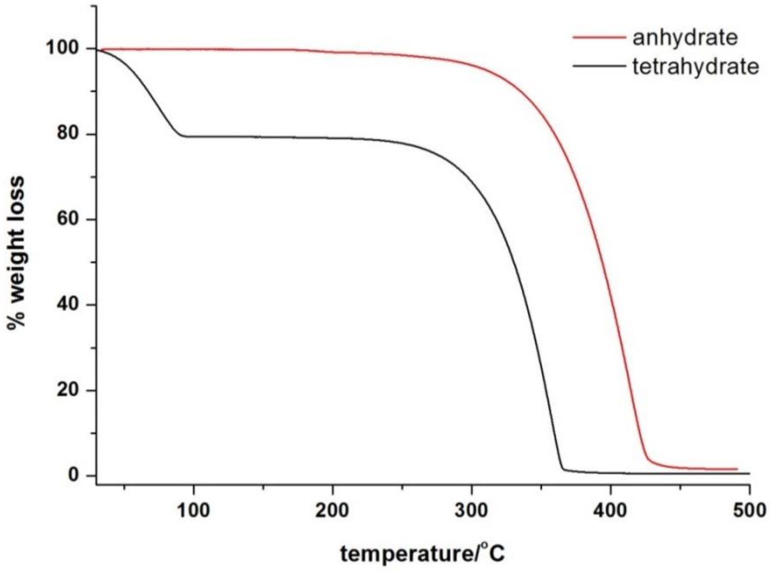

Figure S31 TGA profiles for the anhydrous and hydrated (**7**·4H_2O) forms of **7**.

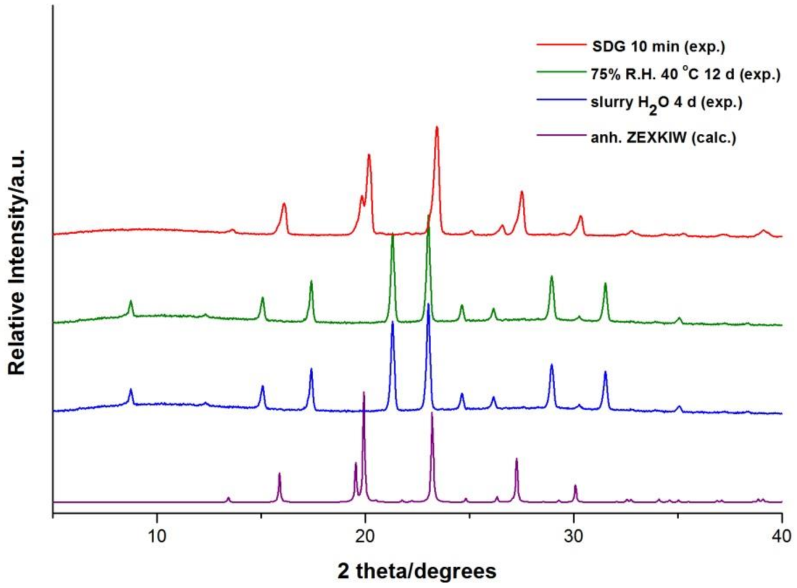

Figure S32 PXRD patterns for anhydrous and hydrated forms of **8**.

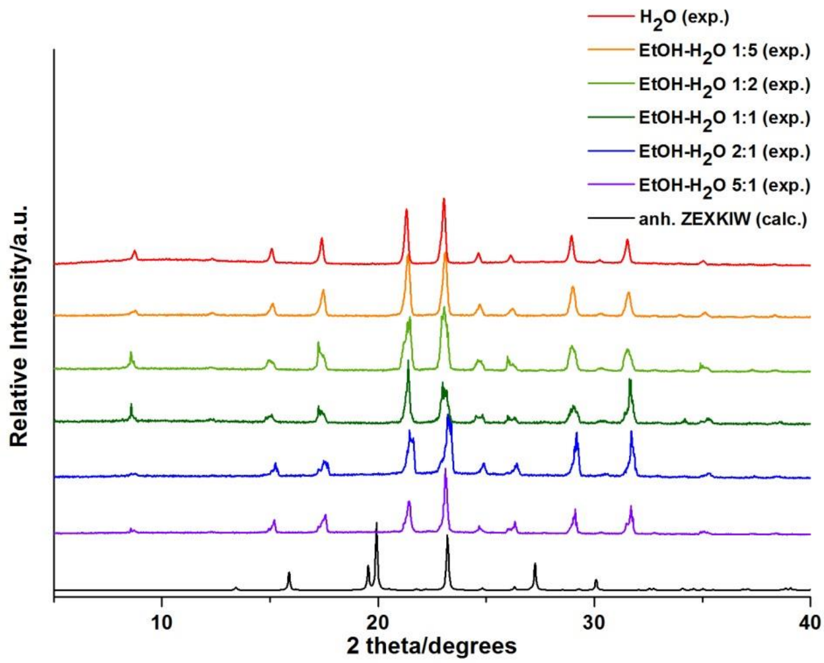

Figure S33 PXRD patterns for competitive slurry experiments for 1:1 w/w mixture of anhydrous and hydrated forms of **8** at rt for 4 d.

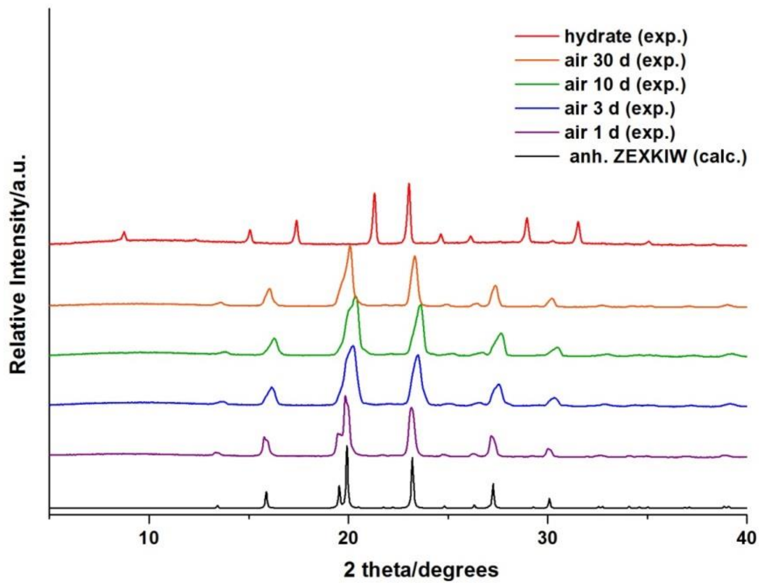

Figure S34 PXRD patterns showing the stability of hydrate form of **8** in air.

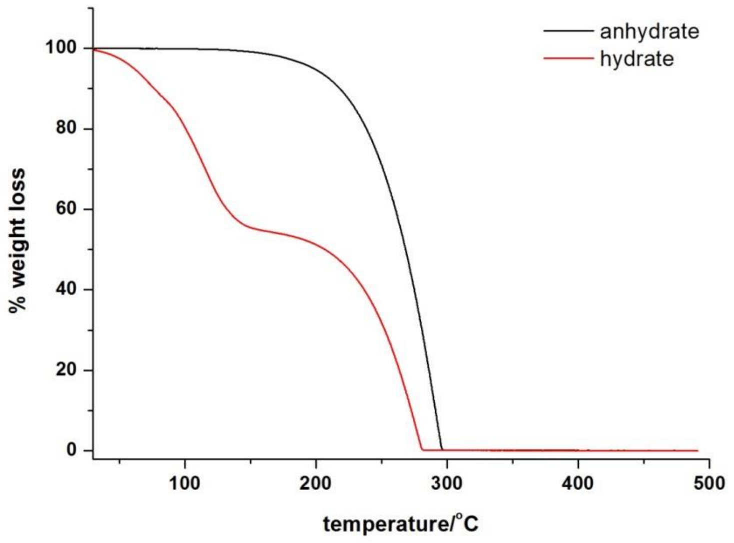

Figure S35 TGA profiles for the anhydrous and hydrated forms of **8**.

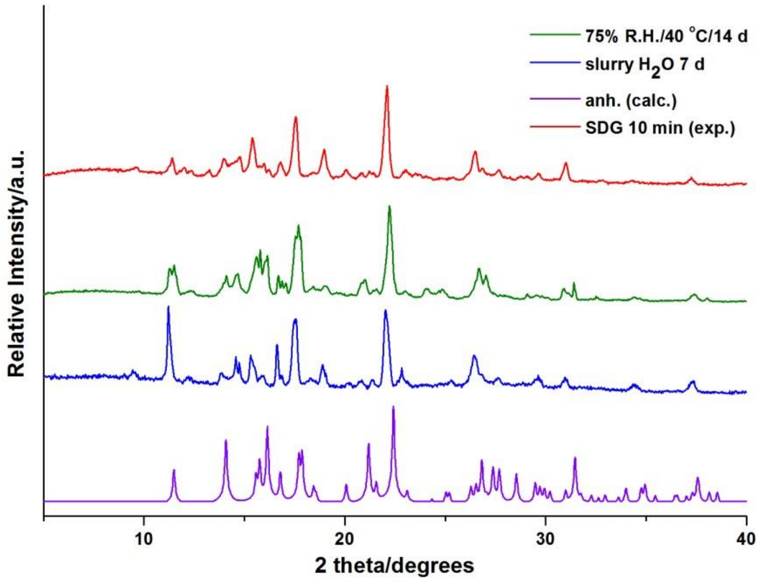

Figure S36 PXRD patterns for anhydrous form of **9** on slurrying in water at rt and exposure to 75% R.H at 40 ˚C.

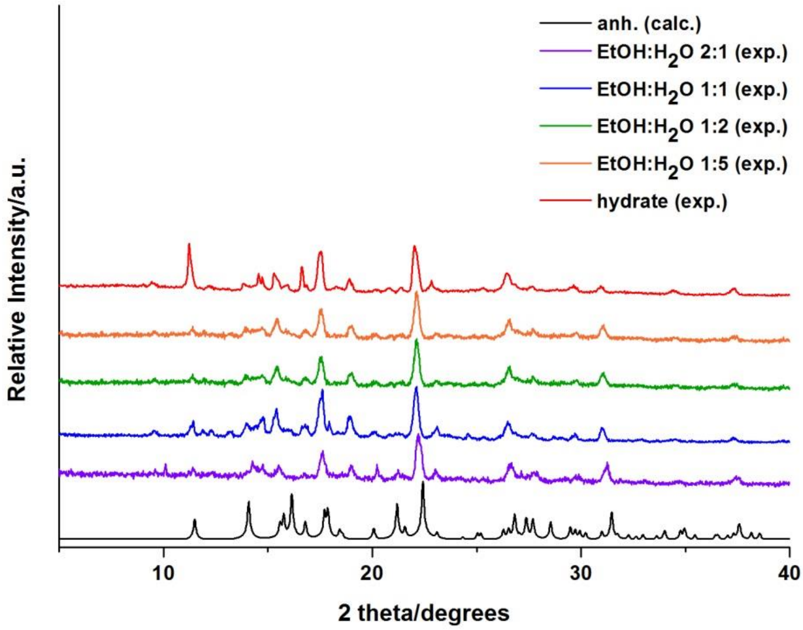

Figure S37 PXRD patterns for competitive slurry experiments for 1:1 w/w mixture of anhydrous and hydrated forms of **9** at rt for 7 d.

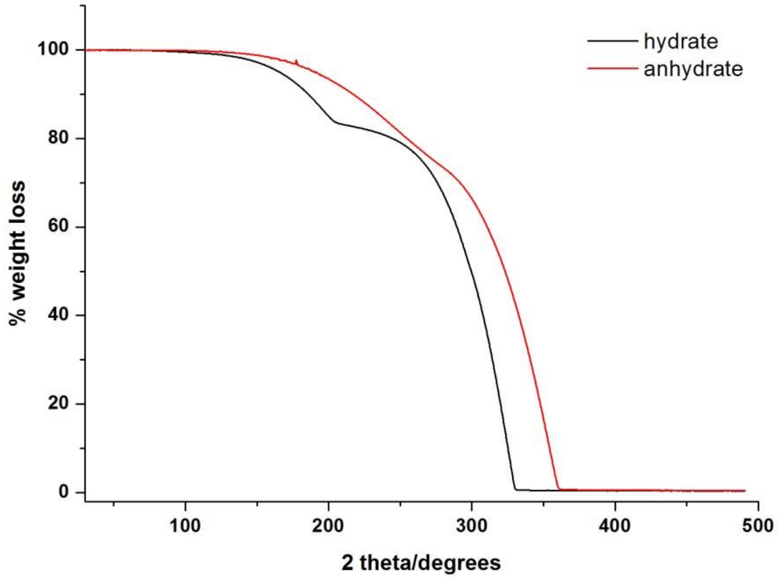

Figure S38 TGA profiles for the anhydrous and hydrated forms of **9**.

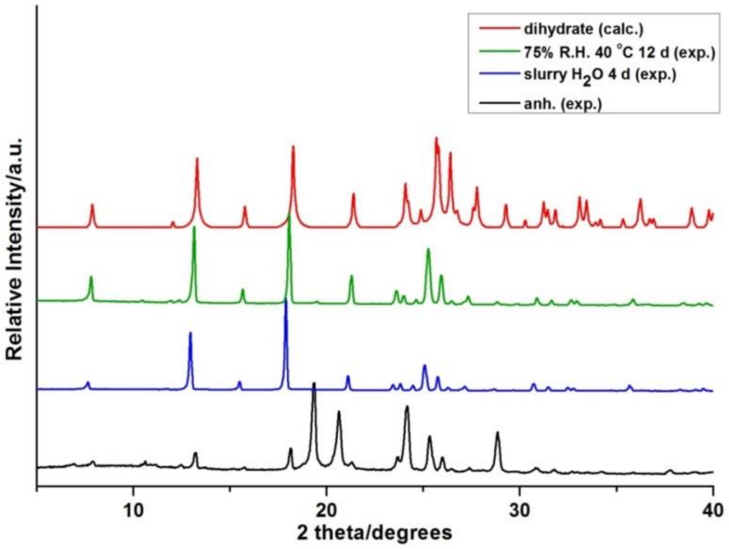

Figure S39 PXRD patterns for anhydrous and hydrated (**10**·2H_2O) forms of **10**.

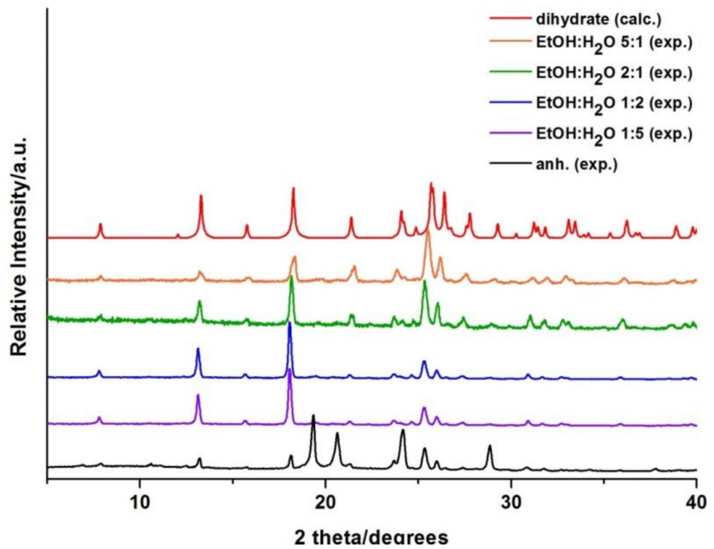

Figure S40 PXRD patterns for competitive slurry experiments for 1:1 w/w mixture of anhydrous and hydrated forms of **10** at rt for 2 d.

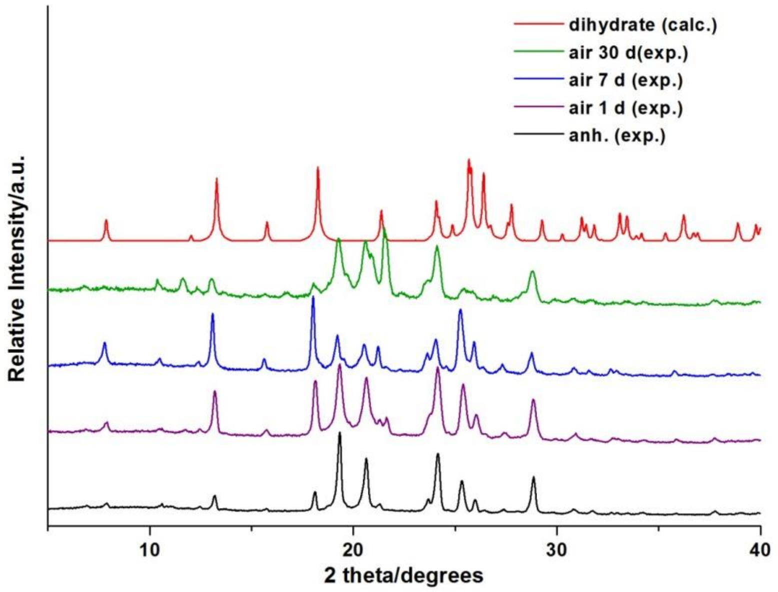

Figure S41 PXRD patterns showing the stability of hydrate form of **10** in air.

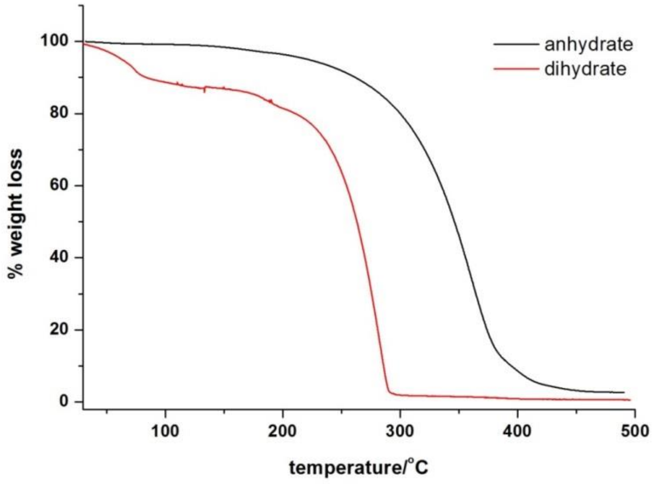

Figure S42 TGA profiles for the anhydrous and hydrated (**10**·2H_2O) forms of **10**.

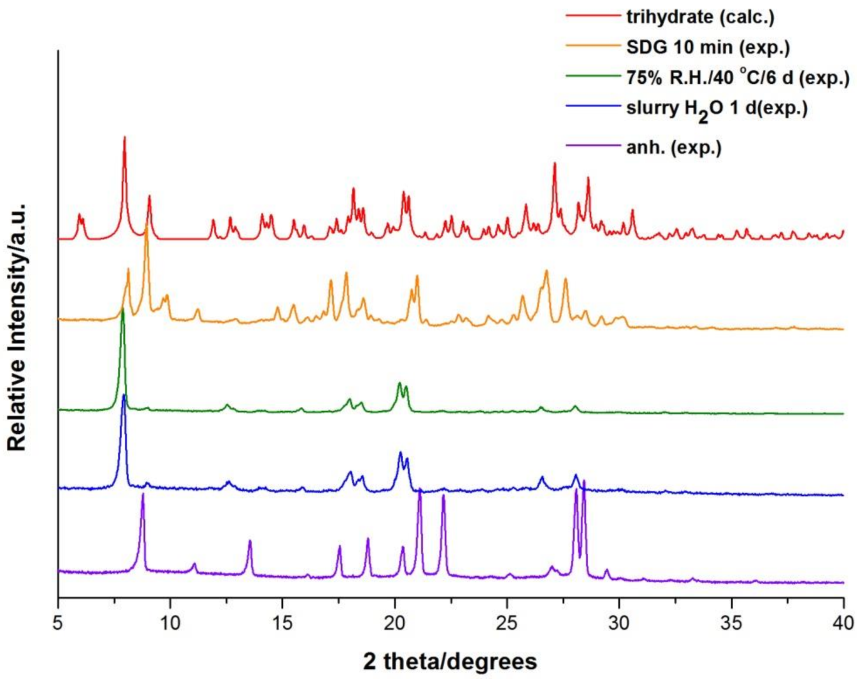

Figure S43 PXRD patterns for anhydrous and hydrated (**11**·3H_2O) forms of **11**.

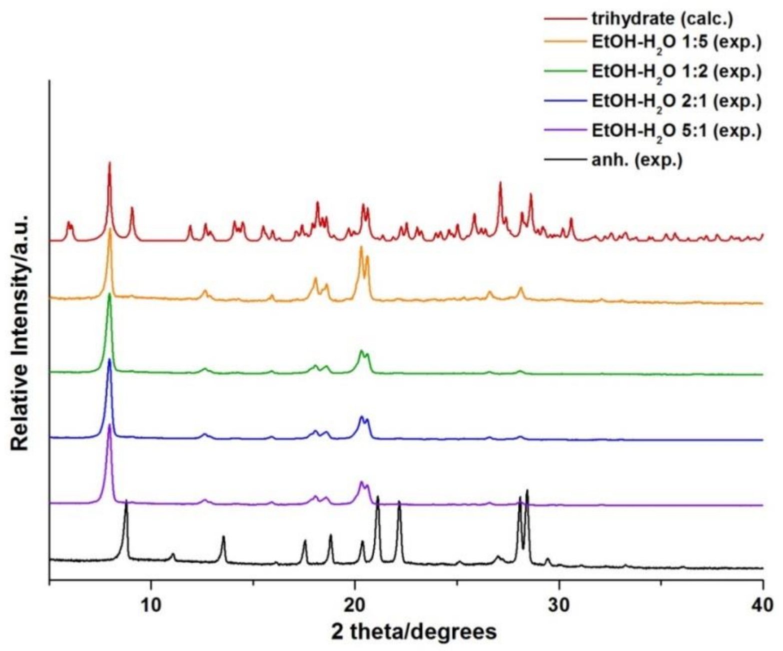

Figure S44 PXRD patterns for competitive slurry experiments for 1:1 w/w mixture of anhydrous and hydrated forms of **11** at rt for 1 d.

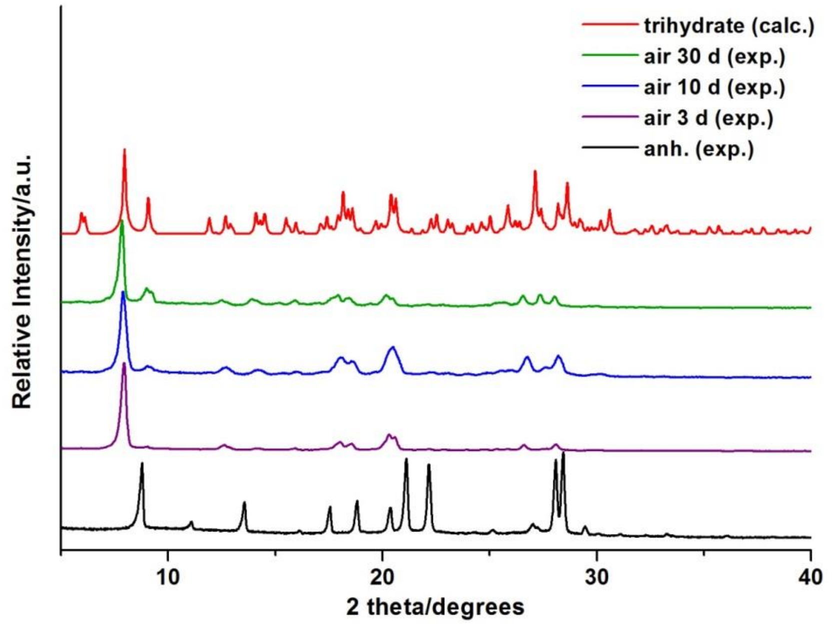

Figure S45 PXRD patterns showing the stability of hydrate form of **11** in air.

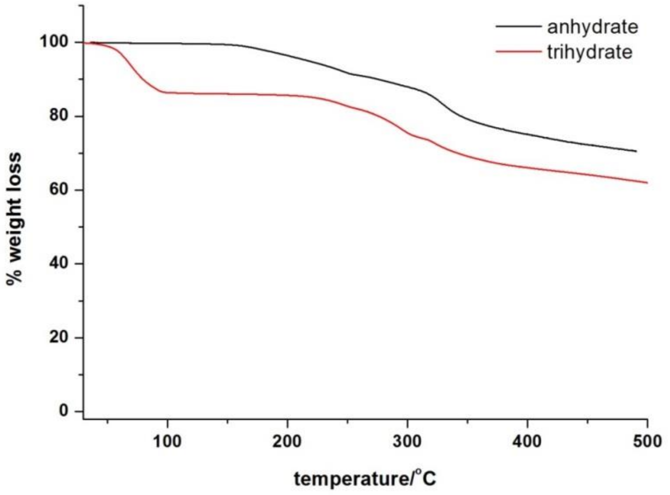

Figure S46 TGA profiles for the anhydrous and hydrated (**11**·3H_2O) forms of **11**.

Table S2. Results of Karl Fisher titration

Compound	Experimental Water Content (%)	Calculated Water Content (%)
4·2H_2O	19.01	18.7
2[a]	0.11	0 (anhydrous)
3[a]	0.44	0 (anhydrous)
9[b]	23.95	23.81 (pentahydrate)

After slurry in water for (a) 5 d or (b) 7 d.
